# Gamma activity concentrations of ^226^Ra, ^232^Th, ^40^K, and health hazard assessments of granites from Wadi El-Nabi’ mining area, Egyptian Nubian Shield

**DOI:** 10.1038/s41598-026-39664-4

**Published:** 2026-02-14

**Authors:** Aya S. Shereif, Mohamed Th. S. Heikal, Abdel Salam Abu El Ela, Ahmed El Shabasy, Ahmed E. Masoud, Árpád Csámer

**Affiliations:** 1 Geology Department, Faculty of Science, Tanta University, Tanta, 31527 Egypt; 2 Department of Mineralogy and Geology, University of Debrecen, Debrecen, 4032 Hungary; 3 The Radiation Protection Department, Nuclear and Radiological Regulatory Authority (ENRRA), Cairo, Egypt; 4 Cosmochemistry and Cosmic Methods Research Group, University of Debrecen, Debrecen, 4032 Hungary

**Keywords:** Gamma index, HPGe detector, Health hazard indices, Wadi El-Nabi’ mining area, Egyptian Nubian Shield, And hydrothermal alteration, Environmental sciences, Natural hazards, Solid Earth sciences

## Abstract

Granitic rocks constitute one of the most prevalent and economically significant lithologies, owing to their abundance, mechanical durability, and aesthetic appeal, which render them highly suitable as ornamental stones in architectural and construction applications. In recent years, extensive research efforts have been directed toward quantifying the radiological hazards posed by naturally occurring radioactive materials within these rocks, concerning their potential implications for human health and environmental safety when utilized in building materials. In the present study, a comprehensive radiometric investigation was conducted on 35 granitic rock samples of Wadi El-Nabi’ mining area, specifically El-Igl El-Ahmer monzo-syenogranites, to quantify the activity concentrations of principal radionuclides, including ^22^^6^Ra, ^232^Th, and ^4^^0^K, utilizing gamma spectrometry with a high-purity germanium (HPGe) detector. Furthermore, an array of radiological hazard indices was systematically calculated to evaluate the potential radiological risks associated with these granitoid specimens. Our findings indicate that the mean activity concentrations of ^226^Ra, ^232^Th, and ^40^K in the monzogranite samples were 29 (± 6), 34 (± 6), and 883 (± 49) Bq/kg, respectively. In comparison, the syenogranite samples exhibited slightly elevated average values, measured at 31 (± 5), 35 (± 4), and 890 (± 9) Bq/kg for the corresponding radionuclides, reflecting a modest enrichment in radioactivity within the syenogranitic lithology. With respect to the radiological parameters, the results indicate that D_out_, D_in_, AEDE_in_, ELCR_out_, ELCR_in_, I_γ_, and AGDE for both monzogranite and syenogranite samples exceed the internationally recommended reference levels. Conversely, Ra_eq_, AEDE_out_, H_ex_, and H_in_ remain within acceptable global thresholds. ^232^Th/^226^Ra (^238^U) ratios for the monzogranite and syenogranite samples range from 0.88 to 1.39 (mean 1.15 ± 0.14) and 0.88 to 1.41 (mean 1.14 ± 0.16), respectively. These values are markedly lower than the canonical crustal Th/Ra ratio of ~ 3.5, indicating post-magmatic hydrothermal alteration and selective uranium enrichment within the host granitoids. This radiological evidence is reinforced by remote sensing observations, which reveal characteristic alteration patterns, including kaolinization, sericitization, fluoritization, and silicification zones, that are spatially associated with the monzo-syenogranitic units, especially in the buffer location. Consequently, the granitic rocks in certain localized areas, particularly where radionuclide concentrations or radiological hazard indices exceed typical thresholds, may be considered unsuitable for use as construction materials.

## Introduction

Naturally occurring radioactive materials (NORMs) are present in various geological formations, including the Earth’s crust, water bodies, soils, and rocks (especially granitoid rocks), which serve as raw materials in building construction. Natural radionuclides are inherently linked to the Earth’s formation and geological processes, making it impossible to eradicate their presence completely. The source of natural radioactivity can be traced by naturally occurring radioisotopes, specifically the ^238^U (^226^Ra) and ^232^Th series, along with their derivatives, and ^40^^K 1^. Collectively termed terrestrial radioisotopes, these elements are distributed widely in minute concentrations throughout the Earth’s crust. These radionuclides are abundant within crustal rocks, certainly the highly evolved granitic rocks, syenites, and pegmatites, linked tomineralogical composition and petrographic features^2^.

Uranium and thorium occur ubiquitously across all lithological units and soil types, yet their spatial distribution within the Earth’s crust is markedly heterogeneous, influenced by geological factors and other variables^3^. This variability arises from a complex interplay of geochemical fractionation and geophysical processes that progressively mobilize, concentrate, and recycle crystalline material between the crust and the mantle^4^. Both thorium and uranium contents tend to be high in felsic rocks and to increase with alkalinity or acidity. Uranium is highly mobile in near-surface environments because it readily oxidizes to soluble uranyl species under oxidizing conditions. This enhanced solubility allows uranium to be efficiently leached from granites, pegmatites, and other felsic rocks, transported significant distances by meteoric or groundwater flow, and subsequently reprecipitated within sedimentary or structurally favorable traps far from its original igneous source. Whereas thorium is relatively stable and much less soluble than uranium and potassium, and does not move except by mechanical means such as wind and erosion processes^5^.

Uranium is a naturally occurring radioactive element, dispersed across several main uranium minerals like uraninite (pitchblende), uranophane, β-uranophane, and U-bearing heavy minerals like monazite, zircon, apatite, columbite, ilmenite, riebeckite, and magnetite, which can contain 61 to 65%^6–8^. Thorium primarily occurs in silicates, oxides, phosphates, and carbonates^2^. In addition, it exists primarily in Th-bearing minerals, like monazite (Ce,La,Nd,Th)PO₄, thorianite (ThO₂), and thorite (ThSiO₄)^4,7–9^. Additional minerals that incorporate substantial thorium include zircon (ZrSiO₄) through Th-substitution for Zr^4^⁺, allanite, xenotime, betafite^8^. Owing to its stronger lithophile behavior and lower mobility under most geological conditions, thorium typically occurs at concentrations approximately three times higher than those of uranium in the majority of crustal rocks^3^. Conversely, potassium manifests in various mineral compositions, notably within potassium feldspathic minerals (alkaline minerals) such as orthoclase, microcline, or in micas, like muscovite and biotite.

Natural radiation primarily arises from two sources: high-energy cosmic ray particles interacting with the Earth’s atmosphere and radioactive nuclides derived from the Earth’s crust. The inherent natural radioactivity of geological materials presents both external and internal radiation hazards in industrial facilities, workplaces, and residential dwellings. These risks arise predominantly from gamma-ray emissions produced by primordial radionuclides, as well as from the inhalation of radon isotopes (^220^Rn and ^222^Rn), which are generated through the radioactive decay of radium (^226^Ra) within the ^238^U decay series. Radon and its short-lived progeny (^218^Po, ^214^Po, and ^214^Bi) emit highly ionizing alpha particles; when these decay products accumulate indoors, particularly in poorly ventilated spaces, they contribute significantly to internal radiation exposure^10–12^. The radiation dose attributable to naturally occurring radionuclides incorporated within building materials, particularly lithological products such as granites under certain circumstances, attain levels of several millisieverts per year (mSv/yr)^13^. Measuring the activity concentrations of radionuclides in building materials is paramount for evaluating population exposure, particularly given that individuals typically spend approximately 80% of their time indoors. Approximately 80% of the overall radiation dose is attributed to natural sources, predominantly stemming from felsic igneous rocks (especially granitic rocks) and soils^14^.

Over recent years, numerous investigations have been undertaken to evaluate the radiological hazards associated with naturally occurring radioactive materials (NORMs) in building materials. The evaluation of the gamma radiation dose from natural sources is particularly important as it is the largest contributor to the external dose^10^. The radiation doses fluctuate according to the levels of naturally occurring radionuclides, such as ^238^U, ^232^Th, their decay products (^208^Tl and ^228^Ac of the ^232^Th disintegration chain, from ^214^Pb and ^214^Bi derived from ^226^Ra of the ^238^U disintegration chain), and ^40^K, present in rocks and soils, which are themselves influenced by the specific geological characteristics of each area^15–17^. Increased and chronic ionizing exposures to radiation levels can lead to the breakdown of living tissues, resulting in significant harm that might eventually lead to the organism’s death. Moreover, even when exposed to lower doses, radiation can heighten the likelihood of cancer development (for example, thyroid, lung, and breast cancers and leukemia). Therefore, exposure to ionizing radiation presents potential risks to both health and the environment, underscoring the importance of careful consideration^14,18–21^.

The Egyptian Precambrian basement complex forms the NW extremity of the Arabian–Nubian Shield (ANS), exposed predominantly throughout the Eastern Desert, southern Sinai, and portions of the SW part of the Western Desert^4^. Within the Eastern Desert, these lithotectonic units constitute a continuous belt extending from the Cairo region in the north to the Sudanese frontier in the south^22^. Granitic rocks constitute the predominant magmatic component of the Egyptian segment of the Nubian Shield, accounting for approximately 60% of the exposed Precambrian basement^22^. Granitic rocks, extensively utilized in construction and architectural applications, ranging from interior, such as decorative aggregates, flooring, and ornamental designs, to exterior functions like building facades, facing stones, and paving materials, carry the potential to elevate radiation exposure due to their natural radioactivity. The use of materials containing elevated concentrations of radionuclides can markedly increase the radiation exposure experienced by the population. Therefore, it is essential to conduct thorough assessments of the radiological risks posed by naturally occurring radionuclides in granite to guarantee safety and adherence to health regulations.

**Fig. 1:**
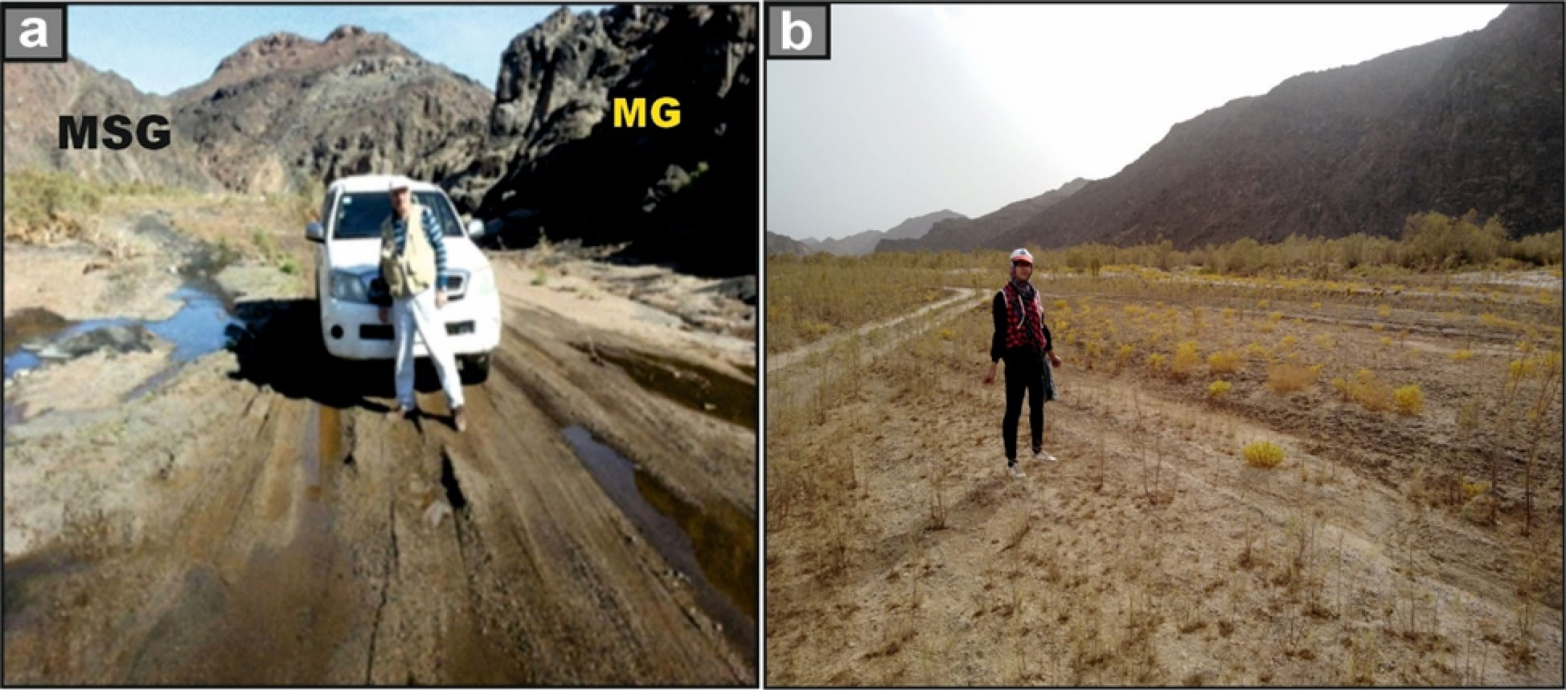
**(a)** Field photographs showing running water, along a desert track of Wadi El-Nabi’, and **(b)** a part of Wadi El-Nabi’, note the dense vegetation cover on both sides of the wadi across the open safari area.

The nomenclature of Wadi El-Nabi’ is attributed to the running water on both sides (Fig. 1a). It is a part of Marsa Alam province, characterized by a very good safari tourist site and an open area to visitors and tourists due to the dense vegetation cover along this wadi and acceptable desert track for driving 4 × 4 cars (Fig. 1b). The studied area has been recognized as a vibrant center for mineral exploration for numerous decades, housing multiple excavation sites focused on extracting various mining and quarrying materials, as evidenced by studies such as those conducted by^23–25^. Huge outcrops of younger monzogranites and syenogranites (El-Igl El-Ahmer Granite) occur on both sides of the present wadi. El-Igl El-Ahmer Granite pluton represents one of the most distinguished rare-metal–enriched granitic intrusions within the Arabian–Nubian Shield (ANS)^26^. Therefore, it is urgent to investigate the radioactivity levels of the studied granitic rocks.

A substantial body of research has been devoted to quantifying natural radioactivity levels and evaluating associated radiological hazard indices within granitic assemblages of the Egyptian Nubian Shield and South Sinai provinces^17,18,24–27^. Despite this extensive regional coverage, the present investigation constitutes the first systematic assessment of radionuclide concentrations in the granitic rocks of the Wadi El-Nabi’ area. To date, no published studies have addressed the radioelement distribution, radioactivity signatures, or radiological implications of the granites from this locality, rendering the current work a novel contribution to the radiological characterization of the region.

The ongoing research focuses on evaluating the levels of natural radionuclides (^226^Ra, (^238^U), ^232^Th, and ^40^K) and analyzing their radioactivity hazard characteristics within the recently formed monzogranitoids and syenogranites obtained from Wadi El-Nabi’ mining region. Furthermore, the study explores potential health risks associated with these findings and compares the obtained results to the recommended limits. The information obtained is crucial for decision-makers and will also serve as valuable groundwork for future inquiries into public health and environmental safety.

## Geology of the study area

**Fig. 2:**
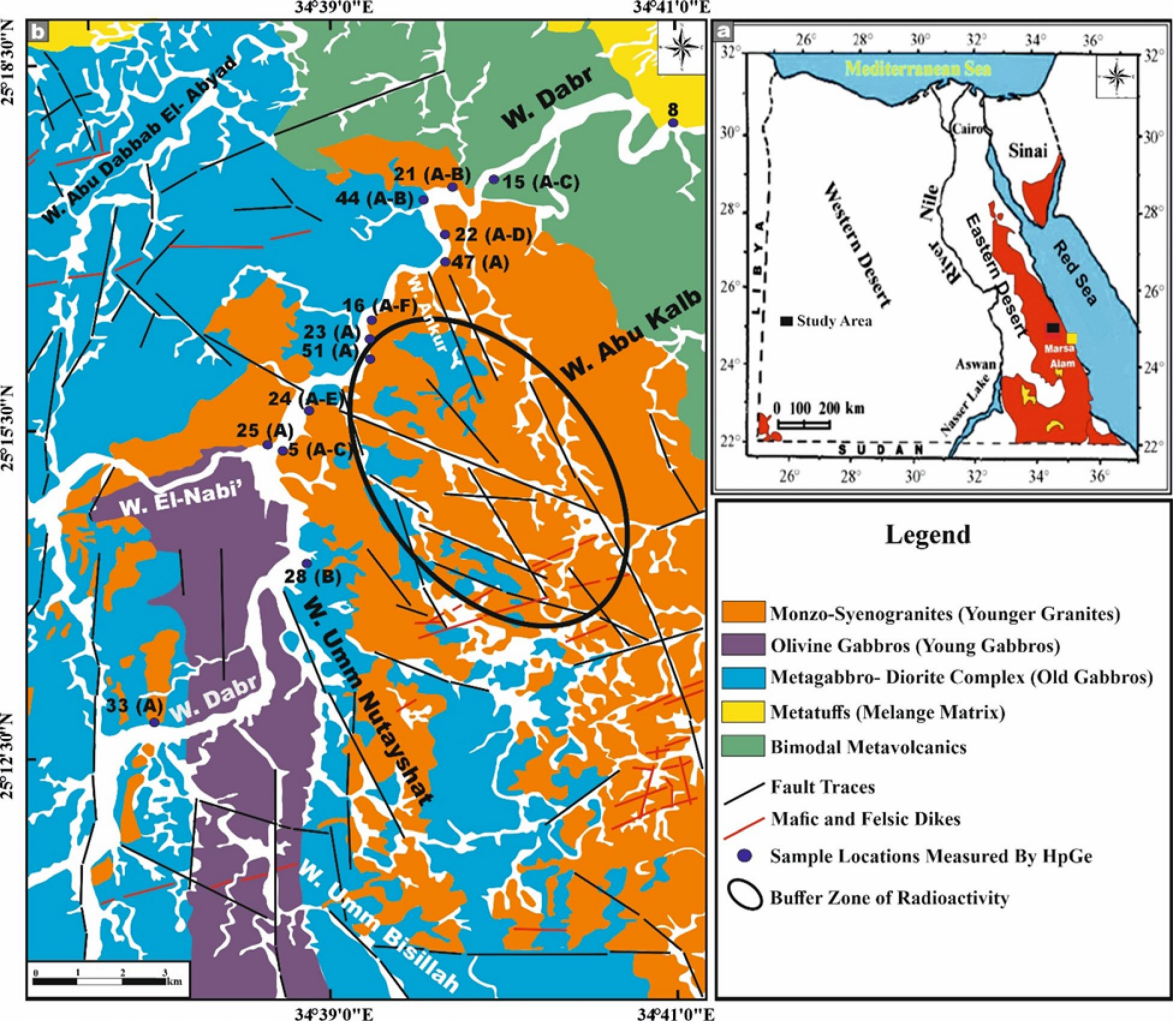
**(a)** Location map showing the study area (black rectangle) within the Egyptian Nubian Shield and **(b)** Geological map of Wadi El-Nabi’ area after^20,25^ (Created by QGIS v. 3.40.9–Bratislava software; https://qgis.org/).

The study area covers approximately 120 km^2^ and is located about 30 km northwest of Marsa Alam town, within the Egyptian Nubian Shield (ENS), which represents the NW part of the Arabian Nubian Shield (Fig. 2). This area has attracted considerable attention from previous researchers^20,25,28,30^, who focused on its geological characteristics and whole-rock geochemistry.

The investigated area constitutes a segment of the Precambrian extrusive–intrusive lithological assemblage. This geologic domain is distinguished by a heterogeneous assemblage of igneous bodies, comprising mafic to felsic metavolcanic sequences and metatuffs, which are transected by swarms of both mafic and felsic dykes (Fig. 2). The metagabbro–diorite complex (older gabbros) and the olivine and hornblende-bearing gabbros, collectively designated as younger gabbros, intrude these volcanic and metatuff units. All of the aforementioned lithologies are subsequently intruded by conspicuously younger monzo-syenogranitic plutons (El-Igl El-Ahmer Granite) (Fig. 2).

**Fig. 3:**
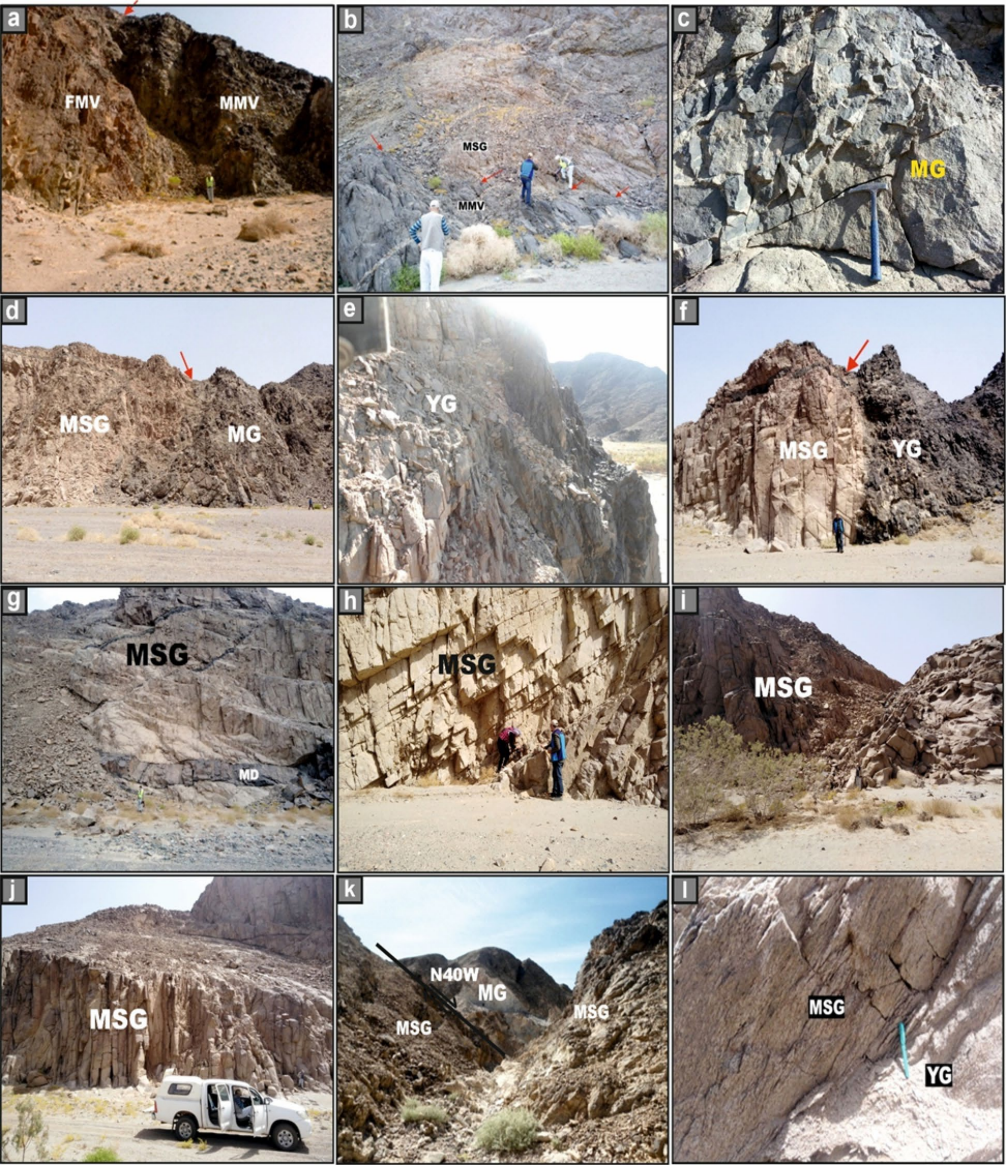
Field photographs capturing the principal geological features of the Wadi El-Nabi’ area. **(a)** sharp contact (red arrow) between the mafic metavolcanics (MMV) and felsic one (FMV), **(b)** sharp intrusive contact (red arrows) between El-Igl El-Ahmar monzo-syenogranites (MSG) and the mafic metavolcanics (MMV), **(c)** massive blocky appearance of an exposure of the metagabbro-diorite complex (MG), **(d)** sharp intrusive contact (red arrow) between monzo-syenogranites (MSG) and the metagabbros (MG), **(e)** highly sheared young gabbros (YG) along intensive shear zone, **(f)** sharp intrusive contact (red arrow) between monzo-syenogranites (MSG) and the young gabbros (YG), **(g)** massive and fresh exposures of El-Igl El-Ahmar monzo-syenogranites (MSG), **(h)** monzo-syenogranitoids exposed as discrete, fault-bounded blocks, **(i-j)** pervasive fracturing and advanced degrees of weathering affecting the younger granites, **(k)** fault trace across the monzo-syenogranites (MSG) and the metagabbros-diorite complex (MG), and **(l)** striations and slicken sided of fault along outcrops of monzo-syenogranites pluton (MSG) and young gabbros (YG). Symbols MD = mafic dyke.

The felsic and mafic metavolcanic rocks, comprising metadacite, metarhyolite, and metabasalt, display well-preserved volcanic structures and characteristic metamorphic overprints. The contact between the mafic and felsic metavolcanic units is clearly discernible in the field (Fig. 3a). The metavolcanic assemblage is bounded to the south by the El-Igl El-Ahmer monzo-syenogranite pluton, with which it forms a distinctly intrusive contact (Figs. 2 and 3b). Metatuffs occupy the N sector of the mapped area and display considerable lithological and textural variability, ranging from fine-grained tuffs to coarse, lapilli-rich varieties (Fig. 2).

The Metagabbro–Diorite Complex comprises a heterogeneous assemblage of metagabbros, diorites, and metadolerites. These rocks exhibit a distinctly blocky morphology and are typically massive, intensely jointed, and pervasively fractured (Fig. 3c). They display characteristic greenish hues and preserve clear evidence of magmatic hybridization and late-stage (deuteric) alteration processes. The complex intrudes the surrounding metavolcanics and metatuffs (Fig. 2). Conversely, the complex is intruded from the east by the El-Igl El-Ahmar monzo-syenogranitic pluton and by younger gabbroic intrusions, both of which establish sharp, irregular intrusive contacts (Figs. 2 and 3d). The young gabbroic mass forms low, rugged hills and varies from dark to greyish green, with grain sizes ranging from fine to coarse (Fig. 3e). Lithologically, it is chiefly composed of olivine gabbro, hornblende gabbro, and troctolite, and occurs as massive, non-layered exposures. This gabbroic body intrudes the metagabbro–diorite complex along sharp contacts and is subsequently cross-cut by the Igl El-Ahmar monzo-syenogranite pluton(Figs. 2 and 3f).

Monzo-syenogranites, covering an area of approximately 55 km^2^, represent a major component of the younger El-Igl El-Ahmar granite, which is regarded as one of Egypt’s fourteen mineralized granitic plutons^31^ (Fig. 2). These rocks are typically medium to coarse-grained and exhibit distinctive pale-pink to pinkish-red colouration. They form the central massif of the study area, where they occur as extensive, massive, and relatively fresh exposures with elevations ranging from 55 to 650 m above sea level (Fig. 3g). In addition to their main outcrops, the monzo-syenogranitoids appear as discrete, fault-bounded blocks that define mountainous terrains predominantly aligned along NW–SE-trending structural corridors (Fig. 3h). The pluton is strongly affected by pervasive fracturing, weathering and intense shearing (Fig. 3i, j). Morphologically, the pluton exhibits an overall elliptical (oval) configuration. Its W sector is distinguished by a prominent roof pendant formed by the tectonic uplift of the young gabbros and metagabbro–diorite rocks during the intrusion (Fig. 2). The boundary between the El-Nabi’ younger monzo-syenogranitoids and the adjacent rock formations is characterized by intrusive relationships, with the monzo-syenogranites enclosing xenoliths and enclaves derived from the surrounding host rocks.

The granitic pluton is dissected by a suite of mafic dykes (basaltic composition) (Fig. 3g) and felsic dykes (aplite and microgranite), which exhibit dominant ENE–WSW and N–S orientations, with the former being notably more prevalent. A subordinate NW–SE trend is also discernible (Fig. 2). In addition, quartz, carbonate, and epidote veins transect the pluton, further attesting to multiple phases of brittle deformation and hydrothermal activity.

Furthermore, the entire lithological framework is extensively transected by a network of brittle–ductile shear zones and fault systems, predominantly oriented along NW–SE and N–S major trends (Figs. 2, 3k–l), with subordinate lineaments exhibiting a NE–SW orientation (Fig. 2).

## Materıals and methods

### Sampling, sample preparation techniques and experimental setup

The quantification of radioactivity in environmental investigations can be accomplished through the deployment of radiation detectors, which encompass a broad spectrum of detection technologies tailored to various radiation types. The fundamental operating principles of these detectors are intrinsically governed by the interaction of ionizing radiation with matter (the detector medium), where distinct interaction processes determine the detector’s efficiency, resolution, and overall performance. In the present study, the essential physics underlying radiation-matter interactions has been elaborated to provide the necessary conceptual framework. For instance, when the sample exhibits heterogeneous compositional characteristics and produces a complex, multi-energy gamma spectrum with numerous overlapping peaks, high-purity germanium (HPGe) detectors are markedly superior to scintillation-based detectors due to their exceptional energy resolution and analytical precision.

Thirty-five representative ground samples were systematically collected from the monzo-syenogranitic assemblages of the El-Igl El-Ahmer pluton, comprising 15 monzogranite and 20 syenogranite specimens. Each sampling station was precisely delineated and documented (Fig. 2). The geographic coordinates of all samples were accurately constrained using an MG-950 CPS unit, ensuring robust spatial referencing.

After carefully removing alteration surfaces, 200 g of each granitic rock sample were thoroughly pulverized into a fine powder and passed through a 1 mm sieve to eliminate coarser particles, ensuring sample homogeneity. This preparation was conducted at the sample preparation laboratory of the Geology Department, Tanta University, Egypt. Subsequently, the powdered samples were dried in a temperature-controlled oven at 110 °C for 24 h to completely remove moisture. Once dried, the samples were stored under controlled laboratory conditions. The accurately weighed powders were then securely packed in polyethylene bags, clearly labeled, and sealed tightly with gas-impermeable parafilm. To establish radioactive equilibrium between ^226^Ra and ^222^Rn, the sealed samples were stored undisturbed for approximately 30 days^85^.

**Fig. 4:**
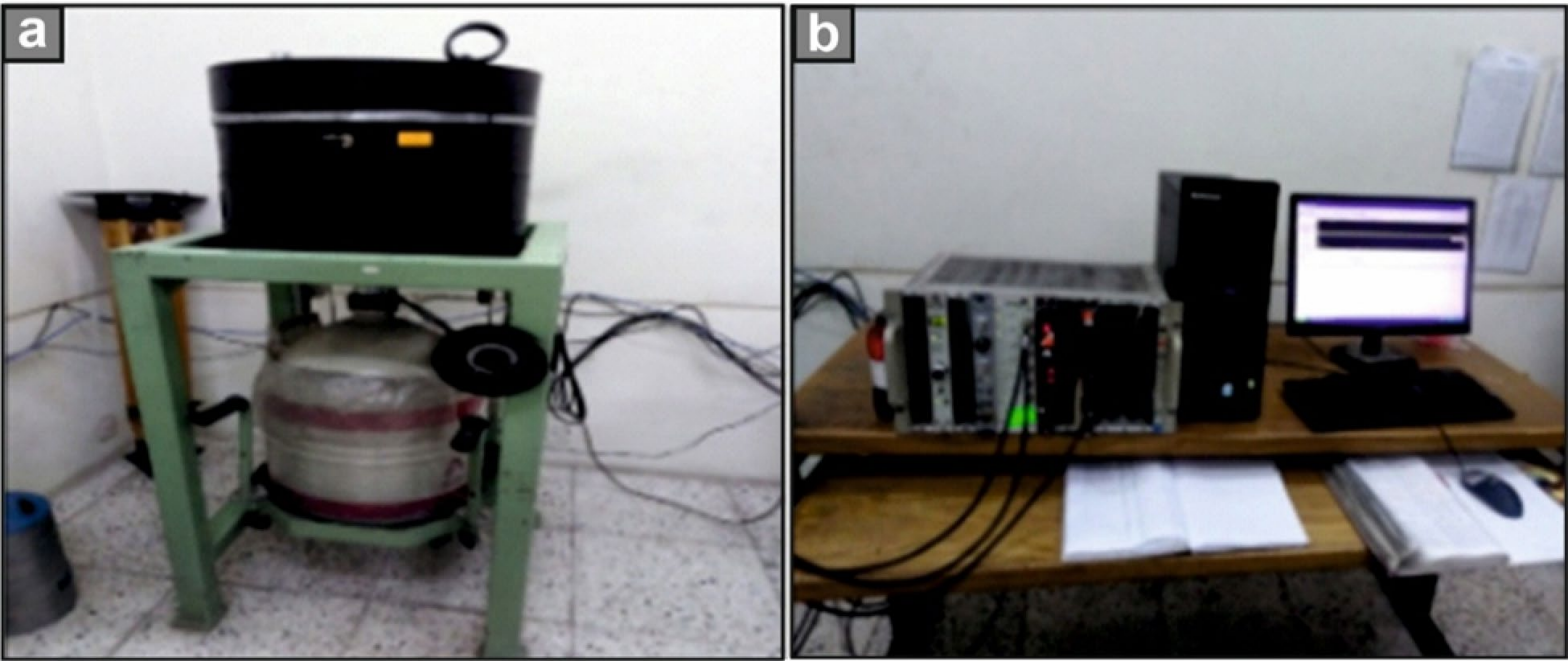
**(a)** Vertical HPGe detector with the lead shield, and **(b)** Canberra Genie 2000 software was used for gamma acquisition, and the analysis system was supported by the LabSOCS (Laboratory Source Less Calibration Software).

- Concurrently, identical empty Marinelli beakers (100 cm^3^ volume) designated for sample storage were sealed and stored under identical conditions alongside the samples. This parallel setup enabled the simultaneous measurement of environmental background radiation levels throughout the storage period. Subsequent gamma spectrometric analysis was conducted at the Radiation Protection Department of Egypt’s Nuclear and Radiological Safety Research Center, Egyptian Atomic Energy Authority, ensuring consistent evaluation of both samples and background controls.
- Sample counting and detector efficiency calibration, the samples were placed into the active volume of a shielded high-purity germanium (HPGe) detector, which had two inner concentric cylinders of lead, copper, and cadmium, as well as its electronic circuits. A vertical Canberra N-type closed-end coaxial Canberra N-type HPGe detector (model GR4020) with about 40% relative efficiency and 2.0 keV energy resolution at 1.33 MeV photons of 60 °C was used. This detector is shielded by a detector lead shield model 747/747E with an Outer Jacket of 9.5 mm (3/8 in.) thick low-carbon steel, a bulk shield of 10 cm (4 in.) thick low background, and a graded lining of 1 mm (0.040 in.) tin and 1.6 mm (0.062 in.) copper. The spectra were analyzed using CANBERRA (Genie 2000) program (Fig. 4a).
- Efficiency Calibration of the Analyzer Channels, the efficiency calibration of the analyzer channels for the HPGe detector is routinely performed using standard point sources, as outlined in the Genie-2000 Spectroscopy Software manual^32^ (Fig. 4b). This process establishes a precise correlation between gamma-ray energy and the corresponding channel number. Following energy identification with standard sources, the detector’s absolute efficiency was calibrated using mixed gamma sources containing multiple radionuclides arranged in the same geometric configuration as the samples. The efficiency values were determined by accounting for the disintegration probability of each gamma energy. This calibration data is essential for accurate detector performance and is mathematically represented as described in *Eq. *(1) ^33^.
  1 $${\upvarepsilon }\left( {{\mathrm{E}}_{{\upgamma }} } \right) = \frac{{{\text{NP }} \times {\mathrm{M}}}}{{{\mathrm{t}}_{{\mathrm{c}}} \times {\mathrm{I}}_{{\upgamma }} \left( {{\mathrm{E}}_{{\upgamma }} } \right) \times {\mathrm{A}}_{{{\mathrm{Ei}}}} }}$$

Where: $\varepsilon \left( {E_{\gamma } } \right)$ is the detection efficiency at energy NP number of counts under the peak for the considered energy corrected for background. The activity concentration (in Bq/kg), AEi of nuclide I, and for the peak at energy E, t is the counting time in sec, $I_{\gamma } \left( {E_{\gamma } } \right)$ the probability of gamma emission of the nuclide for a transition at energy E, and M the mass in kg of the measured sample.

d. Quality assurance was carried out through the analysis of IAEA-381^34^ and IAEA Soil-6^35^ reference materials with a known concentration of natural radioactivity.
e. To determine activity concentration values (AEi) for radionuclide i (in Bq/kg), each sample underwent gamma spectrometric analysis with a counting duration of 72,000 s. Spectral data processing was performed using Genie 2000 spectroscopy software^32^, complemented by Version V.3.2 analytical tools (Fig. 4a, b). The software suite incorporated functionalities such as peak identification, radionuclide characterization, activity quantification, uncertainty estimation, and modules for calculating minimum detectable activity (MDA) using *Eq. *(2) ^36^.
  2 $${\mathrm{A}}_{{{\mathrm{Ei}}}} \left( {\frac{{{\mathrm{Bq}}}}{{{\mathrm{kg}}}}} \right) = \frac{{{\mathrm{NP}}}}{{{\mathrm{t}}_{{\mathrm{c}}} \times {\mathrm{I}}_{{\upgamma }} \left( {{\mathrm{E}}_{{\upgamma }} } \right) \times {\upvarepsilon }\left( {{\mathrm{E}}_{{\upgamma }} } \right) \times {\mathrm{M}}}}$$
  In the uranium decay series, the segment beginning with ^226^Ra holds the greatest radiological significance. Consequently, most studies and references primarily focus on ^226^Ra and its daughter isotopes, as detailed in both our manuscript and numerous published sources. Therefore, our analysis concentrates on the data about ^226^Ra rather than ^238^U. This is because the 1001 keV gamma emission from ^238^U has a very low emission probability (approximately 0.83%), making it less reliable for precise measurements.
g. Under the assumption that secular equilibrium was reached between ^232^Th and ^238^U and their decay products, the γ-ray transitions to measure the concentration of the assigned nuclides in series 1^37^ are as follows: ^234 mPa^ (1001.03 keV) for ^238^U, ^214^Bi (609.31, 1120.3 and 1764.49 keV), ^214^Pb (295.22 and 351.93 keV) for ^226^Ra, ^208^Ti (583.19 and 2614.53 keV), ^212^Pb (238.63 and 300.09 keV) and ^212^Bi (727.3 keV) for the 232Th series, ^228^Ac (338.32, 463.1, 911.20 and 968.97 keV) for ^228^Ra and (1460.83 keV) for ^40^K.
h. To accurately determine the true activity of the sample, statistical error calculations are performed during the measurement process, accompanied by essential corrections applied to the observed count rate. These corrections typically account for factors such as detector efficiency, the emission probability of the specific radiation, the net peak counts (NP), and the sample mass. The overall uncertainty in the calculated activity is then evaluated using the error propagation formula as outlined in Eq. (3) ^38^.
  3 $$\Delta A_{Ei} = A_{Ei} \times \sqrt {\left( {\frac{\Delta M}{M}} \right)^{2} + \left( {\frac{\Delta NP}{{NP}}} \right)^{2} + \left( {\frac{{\Delta I_{\gamma } \left( {E_{\gamma } } \right)}}{{I_{\gamma } \left( {E_{\gamma } } \right)}}} \right)^{2} + \left( {\frac{{\Delta t_{c} }}{{t_{c} }}} \right)^{2} + \left( {\frac{{\Delta \varepsilon \left( {E_{\gamma } } \right)}}{{\varepsilon \left( {E_{\gamma } } \right)}}} \right)^{2} }$$
  The activity concentration of ^238^U is typically estimated from the gamma emissions of its radon progeny, based on the assumption of secular equilibrium among the daughter radionuclides. This assumption holds only under undisturbed conditions where a natural secular equilibrium exists between ^238^U and ^226^Ra. However, under oxidizing environments, uranium predominantly exists in its hexavalent state, making it more prone to mobility and loss compared to radium. In such situations, the 63.29 keV gamma emission from ^234^Th provides the most reliable direct gamma-ray signature for accurately determining ^238^U activity through gamma-ray spectrometry ^39^.
j. Thorium is highly insoluble and therefore thorium concentrations cannot be measured from the gamma lines of the thoron daughters in cases of disequilibrium between ^224^Ra and thoron ^220^Rn daughters. In this case, the two important gamma energies of ^228^Ac, the third member of the series, 338 keV (11.3%), and 911 keV (25.8%), are often used to predict the parent thorium^40^. Thus, the specific activity calculations of the ^238^U and ^232^Th series were obtained indirectly from the gamma rays emitted from their progenies, assuming secular equilibrium, while the ^40^K activities were determined from the 1460.7 keV gamma line.

### Remote sensing data

Landsat-9, the most recent and technologically refined platform in the Landsat satellite lineage, is equipped with a suite of advanced sensor capabilities specifically engineered to support a wide spectrum of remote-sensing applications. This satellite imagery was employed as a robust and efficient tool for improving lithological discrimination and the cartographic delineation of the granitic rock units^41^. Moreover, it proved highly effective in detecting and spatially characterizing hydrothermal alteration zones associated with radioactive mineralization hosted within the younger granitic suites. In our study, Landsat-9 satellite imagery was meticulously processed using QGIS v. 3.40.9–Bratislava software (https://qgis.org/) and ArcGIS Desktop 10.8. (https://www.esri.com/en-us/arcgis/products/arcgis-desktop/overview), seeking to identify Neoproterozoic rocks containing radionuclides within the study area. The image-processing workflow generated several analytical products, including enhanced false-color composites, principal component transformation, and band ratio images at a detailed scale of 1:50,000. These remote sensing products significantly enhanced the geological interpretation by revealing key lithological contacts, structural features, and spatial patterns of hydrothermal alteration, thereby facilitating the accurate delineation of radionuclide-bearing rocks^42–44^.

### Petrographic description

####  Monzogranite

**Fig. 5:**
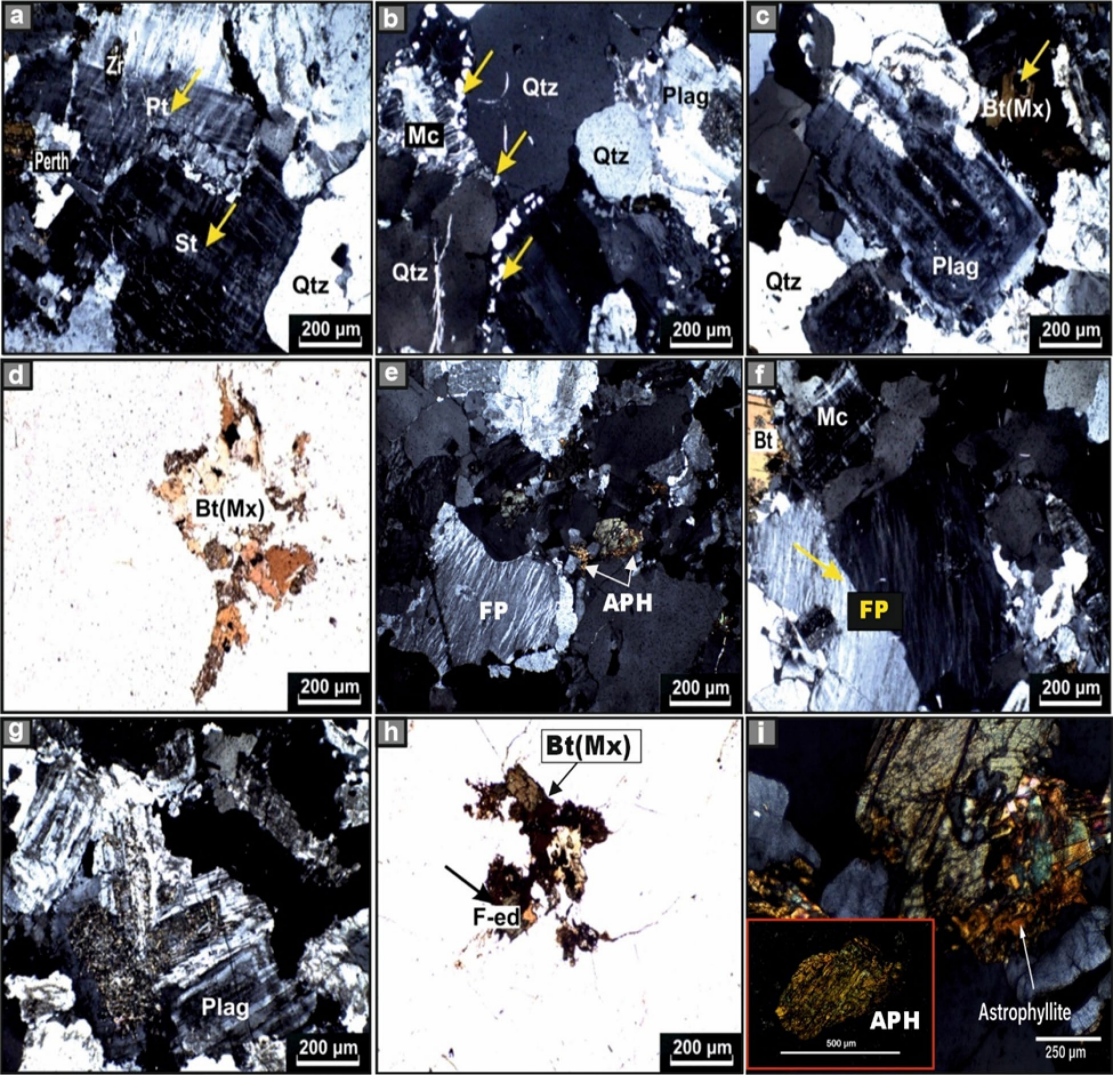
Cross-polarized (XPL) and plane-polarized (PPl) photomicrographs of monzogranite. **(a)** microperthite of string and patch types in association with microcline and quartz, **(b)** anhedral crystals of quartz in association with minute blebs replacing alkali feldspar, **(c)** typical normal zoning in plagioclase in combination with quartz and biotite, **(d)** numerous aggregates of meroxene (biotite). Cross-polarized (XPL) and plane polarized (PPl) photomicrographs of syenogranites. **(e)** typical flame perthitic type in association with quartz and astrophyllite, **(f)** resorption textures in potassic feldspar produced by interaction with adjacent quartz and microcline**, (g)** multi-stages of normal zoning have different directions, extensively altered into kaolinite, **(h)** ferro-edenite locks like wedge-shaped in association with quartz and feldspar, and **(i)** enlarged cross-polarized photomicrograph highlighting the distinctive pleochroism and bladed morphology of astrophyllite. Symbols inside the photos Qtz = Quartz, Pt = Patchy microperthite, St = string microperthite, Perth = Perthite, Plag = Plagioclase, Zr = Zircon, Bt = Biotite, Mx = Meroxene, Mc = Microcline, FP = flame perthite, Asp = Astrophyllite, and F-ed = Ferro-edenite.

These rocks exhibit a medium-grained hypidiomorphic texture and display homogeneous mineralogical and textural characteristics. The primary constituents include potash feldspar, quartz, and plagioclase, accompanied by subordinate Mg-biotite (meroxene) and secondary minerals such as chlorite, muscovite, sericite, kaolinite, and opaques. Common accessory minerals found in these rocks are zircon, apatite, and sphene. The potash feldspar is predominantly composed of microcline-microperthite, with less frequent occurrences of orthoclase-microperthite forming irregular masses and plates (Fig. 5a). Quartz occurs as anhedral to subhedral grains, often interstitial to other mineral phases. It frequently exhibits corrosion and partial resorption of adjacent feldspars, forming circular embayments within them. In some instances, quartz may undergo replacement by surrounding microcline–microperthite, producing bleb-like remnants of quartz within the alkali feldspar (Fig. 5b). Several large quartz crystals have undergone fragmentation into smaller grains, frequently exhibiting features such as strain shadows, glide planes, and characteristic wavy or undulose extinction patterns (Fig. 5b). The plagioclase, identified as albite-oligoclase (An_9-28_), occurs as subhedral, tabular crystals. Slightly altered plagioclase crystals typically show normal zoning patterns (Fig. 5c), while others reveal deformation features including strain shadows, microcracks, and glide planes. Biotite, in its meroxene form, appears as robust flakes with pronounced pleochroism, displaying colors ranging from reddish-yellow along the X-axis to dark brown along the Y and Z axes. This mineral exhibits varying degrees of alteration, often intergrown with chlorite along cleavage planes (Fig. 5c, d). Notably, zircon grains enclosed within these minerals are surrounded by distinctive radioactive halos.

#### Syenogranites

Under microscopic examination, the rock is primarily composed of microcline-microperthite, quartz, plagioclase, and ferro-edenite, with minor amounts of biotite and astrophyllite. Accessory minerals include zircon, apatite, and sphene, while secondary alteration products consist of allanite, chlorite, sericite, and kaolinite. The potash feldspar, identified as microcline-microperthite, occurs as subhedral tabular crystals up to 5 mm in size, predominantly exhibiting a well-developed microperthitic texture (Fig. 5e). These crystals frequently show significant corrosion and resorption features, particularly caused by interactions with quartz and microcline (Fig. 5f). Quartz is observed as large anhedral crystals, forming semicircular embayments and occupying interstitial spaces of up to 9 mm across. The presence of strain shadows leads to wavy and undulose extinction, accompanied by granulation around the borders of other mineral constituents. Plagioclase, characterized by a sodic oligoclase composition (An_14_), manifests as subhedral tabular crystals reaching sizes of up to 5 mm across. Zoning is not intricate, and alteration varies from low to intensive, transitioning into kaolinite within the core and marginal portions of the crystals (Fig. 5g). Chloritization is distinctly evident, forming fibrous aggregates. Biotite (meroxene) is present as a minor component, appearing in the form of clots and fine aggregates in contact with ferro-edenite (Fig. 5h). Astrophyllite, occurring as minor shapeless crystals measuring up to 0.5 mm across, is replete with inclusions (Fig. 5e, i).

## Calculation of radiological hazard parameters

### Radium equivalent activity (Ra_eq_)

Globally, granitoid rocks assume a vital role in construction and building initiatives. The assessment of their intrinsic natural radioactivity centers on scrutinizing the content of radioisotopes like U, Ra, Th, and K. Profound comprehension of these radioisotope levels is imperative to uphold safety standards and adhere to regulations governing construction materials in a variety of construction projects worldwide. Numerous metrics are employed to appraise the gamma radiation risks stemming from radioisotopes, and a noteworthy measure is the radium equivalent activity index (Ra_eq_).

Radium equivalent activity (Ra_eq_) has been practiced for the last ~ 40 years to compare the specific activities of materials containing various levels of ^226^Ra, ^232^Th, and ^40^K. As the latter are unequally distributed in terrestrial materials due to a lack of equilibrium between ^226^Ra and its progenies. It is a weighted sum of the activities of the radionuclides based on the assumption that 10 Bq/kg of ^226^Ra, 7 Bq/kg of ^232^Th, and 130 Bq/kg of ^4^^0^K produce the same gamma dose rates^45^.

4 $$Ra_{eq} = C_{Ra} + \frac{{10C_{Th} }}{7} + \frac{{10C_{K} }}{130} \le 370$$

where, C_Ra_, C_Th_, and C_K_ are the specific activity concentrations of ^226^Ra, ^232^Th, and ^4^^0^K, respectively. The maximum Raeq value detected in granitic rock samples should stay below the recommended safety limit of 370 Bq kg^-1^, as per the guidelines set by^46^.

### Gamma absorbed dose rate in air (D)

The dose rate for absorbed gamma radiation is the amount of ionizing radiation energy absorbed per unit mass per unit time for the material, measured in Gray (Gy h^-1^). The absorbed dose rate in air (ADR) is intricately linked to the activity concentrations of ^226^Ra, ^232^Th, and ^4^^0^K, as these natural radionuclides significantly influence and damage sensitive tissues and organs and contribute to the overall radiation exposure. it can be calculated at 1 m above ground level^36,37^.

5 $${\mathbf{D}}_{{{\mathbf{out}}}} \left( {\frac{{{\mathrm{nGy}}}}{{\mathrm{h}}}} \right) = \left( {0.436{\mathrm{C}}_{{{\mathrm{Ra}}}} } \right) + \left( {0.599{\mathrm{C}}_{{{\mathrm{Th}}}} } \right) + \left( {0.0417{\mathrm{C}}_{{\mathrm{K}}} } \right)$$

6 $${ } {\mathbf{D}}_{in} \left( {\frac{{{\mathrm{nGy}}}}{{\mathrm{h}}}} \right) = \left( {0.92{\mathrm{C}}_{{{\mathrm{Ra}}}} } \right) + \left( {1.1{\mathrm{C}}_{{{\mathrm{Th}}}} } \right) + \left( {0.082{\mathrm{C}}_{{\mathrm{K}}} } \right)$$

where C_Ra_, C_Th,_ and C_K_ are the specific activity concentrations of ^226^Ra, ^232^Th, and ^4^^0^K, respectively.

D_out_: outdoor absorbed gamma dose rate (as a result of terrestrial gamma rays) and D_in_: indoor absorbed gamma dose rate.

For our safety, it is crucial to adhere to the specified maximum limits for D_out_ and D_in_ values, which should not surpass the world averages of 54 and 84 nGy/h, respectively, as outlined by^10^.

### Activity utilization index (AUI )

Building materials play a dual role in radiation dynamics, emitting radiation themselves while also serving as shields against outdoor radiation. This crucial phenomenon, emphasized in the UNSCEAR 2000 report^10^, leads to the effective absorption of radiation emitted by external sources by the walls of buildings. Consequently, indoor air dose rates often exceed the natural radionuclide concentrations present in these materials. The development of AUI was driven by the precise objective of streamlining the computation of air dose rates arising from diverse combinations of three pivotal nuclides, ^226^Ra, ^232^Th, and ^4^^0^K. This index is calculated by an equation, developed by^47,48^, as:

7 $$AUI = \, \left[ {\left( {C_{Ra} /50} \right) * f_{Ra} + \left( {C_{Th} /50} \right) * f_{Th} + \left( {C_{K} /500} \right) * f_{K} } \right] \, < 1$$

where, C_Ra_, C_Th_, and C_K_ are the specific activity concentrations of ^226^Ra, ^232^Th, and ^4^^0^K, respectively. f_Ra_ = 0.462, f_Th_ = 0.604, and f_K_ = 0.041 are the fractional contributions to the total dose rate in the air due to gamma radiation from the pivotal nuclides^49,50^. It is crucial to emphasize that, as per the European Commission 1999^37^, the AUI values must strictly adhere to AUI < 1 to guarantee compliance with safety standards.

###  Annual effective dose rate (AEDE)

Recent research findings reveal that adults typically allocate around 80% of their time indoors, exposing themselves to ionizing radiation emanating from radionuclides present in building materials. Conversely, the remaining portion of their time is spent outdoors, where they encounter radiation from the surrounding environment.

Annual indoor and outdoor effective dose equivalents (AEDE_in_ and AEDE_out_) can be calculated by:

8 $$AEDE_{out} \left( {mSv/year} \right) = D_{out} \left( \frac{nGy}{h} \right) \times \, 8760 \, \left( {h/year} \right) \, \times \, 0.2 \, \times \, 0.7 \, \left( {Sv/Gy} \right) \times \, 10^{ - 6}$$

9 $$AEDE_{in} \left( {mSv/year} \right) = D_{in} \left( \frac{nGy}{h} \right) \, \times \, 8760 \, \left( {h/year} \right) \, \times \, 0.8 \, \times \, 0.7 \, \left( {Sv/Gy} \right) \times \, 10^{ - 6}$$

where 0.7 Sv/Gy was used for the conversion coefficient from the absorbed dose in the air to the effective dose received by adults, and 0.2 and 0.8 are occupancy factors^10^. The annual effective dose equivalent (AEDE) attributable to terrestrial gamma radiation, encompassing both outdoor and indoor sources, stands at a world average of 0.48 mSv/year, according to the findings presented by^10^.

### ***External and internal hazard indexes (H***_***ex***_*** and H***_***in***_***)***

The external hazard index (H_ex_) serves as a crucial evaluation of the risk associated with natural gamma radiation that is emitted from radionuclide elements in soils, rocks, and plants. It functions as a key metric, gauging the relationship that signifies the only external exposure factor of human with their physical environment. It is defined as follows^46^

10 $$H_{ex} = \frac{{C_{Ra} }}{370} + \frac{{C_{Th} }}{259} + \frac{{C_{K} }}{4810} \le 1$$

where C_Ra_, C_Th_, and C_K_ are the specific activity concentrations of ^226^Ra, ^232^Th, and ^4^^0^K, respectively. To minimize the risk of radiation and make exposure insignificant, it is essential to ensure that the Hex value stays below unity.

Alongside the external hazard index (H_ex_), evaluating the internal exposure to radon gas and its decay products requires calculating the internal hazard index (H_in_). Based on the formulation presented by Beretka and Mathew (1985), this index offers a crucial measure of the potential risk posed by alpha radiation to vulnerable internal organs originating from inhaled radioactive materials.

11 $$H_{in} = \frac{{C_{Ra} }}{185} + \frac{{C_{Th} }}{259} + \frac{{C_{K} }}{4810} \le 1$$

To ensure the safe utilization of granitic rocks as a building material, both H_ex_ and H_in_ values should be maintained below unity (< 1), as suggested by^51,52^.

### Excess lifetime cancer risk (ELCR)

Persistent exposure to harmful substances throughout an individual’s lifetime increases the likelihood of cancer development due to exposure to ionizing radiation emitted by radionuclides^53^. As a result, it became essential for us to evaluate the potential carcinogenic effects in a population over a specified lifespan. This process includes predicting anticipated substance intakes and incorporating data related to the chemical’s dose response.

Excess lifetime cancer risk (ELCR) can be calculated through the use of the following expressions:

12 $$ELCR_{out} = \, AEDE_{out} x \, Average \, duration \, of \, life \, \left( {DL} \right) \, x \, Risk \, factor \, \left( {RF} \right)$$

13 $$ELCR_{in} = \, AEDE_{in} x \, Average \, duration \, of \, life \, \left( {DL} \right) \, x \, Risk \, factor \, \left( {RF} \right)$$

where AEDR, DL, and RF are the annual effective dose rate, duration of life (70 years), and fatal risk factor (0.05 Sv^−1^) (i.e., fatal cancer risk per sievert) in the case of stochastic effects^54,55^, respectively.

### ***Representative gamma index (I***_***γ***_***)***

The representative level index (I_γ_) is utilized to evaluate the extent of gamma radiation hazard associated with naturally occurring radionuclides present in the analyzed samples, as defined by^56^. Additionally, the gamma index serves to establish a correlation with the annual dose rate resulting from excess external gamma radiation attributed to superficial materials. It functions as a screening tool to identify materials that could pose health concerns when utilized in construction, as highlighted in the study by^57^.

(I_γ_) can be calculated through the use of the following expressions:

14 $$I_{\gamma } = \frac{{C_{Ra} }}{150} + \frac{{C_{Th} }}{100} + \frac{{C_{K} }}{1500}$$

where C_Ra_, C_Th_, and C_K_ are the specific activity concentrations of ^226^Ra, ^232^Th, and ^4^^0^K, respectively. Values should correspond to an annual effective dose of less than or equal to 1 mSv (I_γ_ ≤ 1).

### ***Alpha index (I***_***α***_***)***

The alpha or internal index (I_α_) serves as a crucial parameter for assessing the level of excess α-radiation that occupants of buildings may be exposed to. This radiation typically originates from radon gas (^222^Rn), which is emitted from materials commonly used in the construction of residential buildings. This standard is established to mitigate potential health risks associated with radiation exposure^57^. The methodology outlined by^58^ and the equation that is employed in the calculation of I_α_ is:

Where C_Ra_ is the specific activity concentration of ^226^Ra. To ensure the safety of buildings for human habitation, the activity of ^226^Ra must remain below the upper limit of 200 Bq/kg. [not exceed the exemption limit of 0.5 Bq/Kg]^59^.

###  Annual gonadal dose equivalent (AGDE)

The annual gonadal dose equivalent (AGDE) and the repercussions of radiation exposure on numerous critical organs, such as the female breast, bone marrow, thyroid, lungs, and reproductive organs (gonads), have garnered significant attention. When these organs are subjected to ionizing radiation, even small doses gradually accumulate within them. Over time, these fractional amounts can reach levels that pose harm, potentially leading to damage and adverse health effects^10,60^. The gonads, active bone marrow, and bone surface cells are recognized as critical organs due to their biological functions and sensitivity. The Annual Genetic Dose Equivalent (AGDE) serves as an indicator reflecting the genetic impact of the annual radiation dose absorbed by rapidly proliferating cells within these radiosensitive organs across a given population.

The annual gonadal dose equivalent (AGDE) due to the specific activities of ^238^U, ^232^Th, and ^4^^0^K is computed using the following:

16 $$AGDE \, \mu Sv \, y^{ - 1} = \, 3.09C_{Ra} + 4.18C_{Th} + 0.314C_{K}$$

The effective dose rate delivered to a specific organ (D_organ_) can be calculated using the following relation^54,61^.

17 $$D_{organ} = AEDE \times F$$

where D_organ_ is the effective dose rate to the organs, AEDE represents the annual effective dose equivalent, and F is the conversion factor of organ dose from air dose.

The average values of the (F) for different organs or tissues according to^62^ are lungs 0.64, ovaries 0.58, bone marrow 0.69, testes 0.82, and the entire body 0.68.

## Results and discussion

### ***Distribution of radioisotope activity concentrations (***^***226***^***Ra, ***^***232***^***Th***, and ^4^^0^K ***)***

**Fig. 6:**
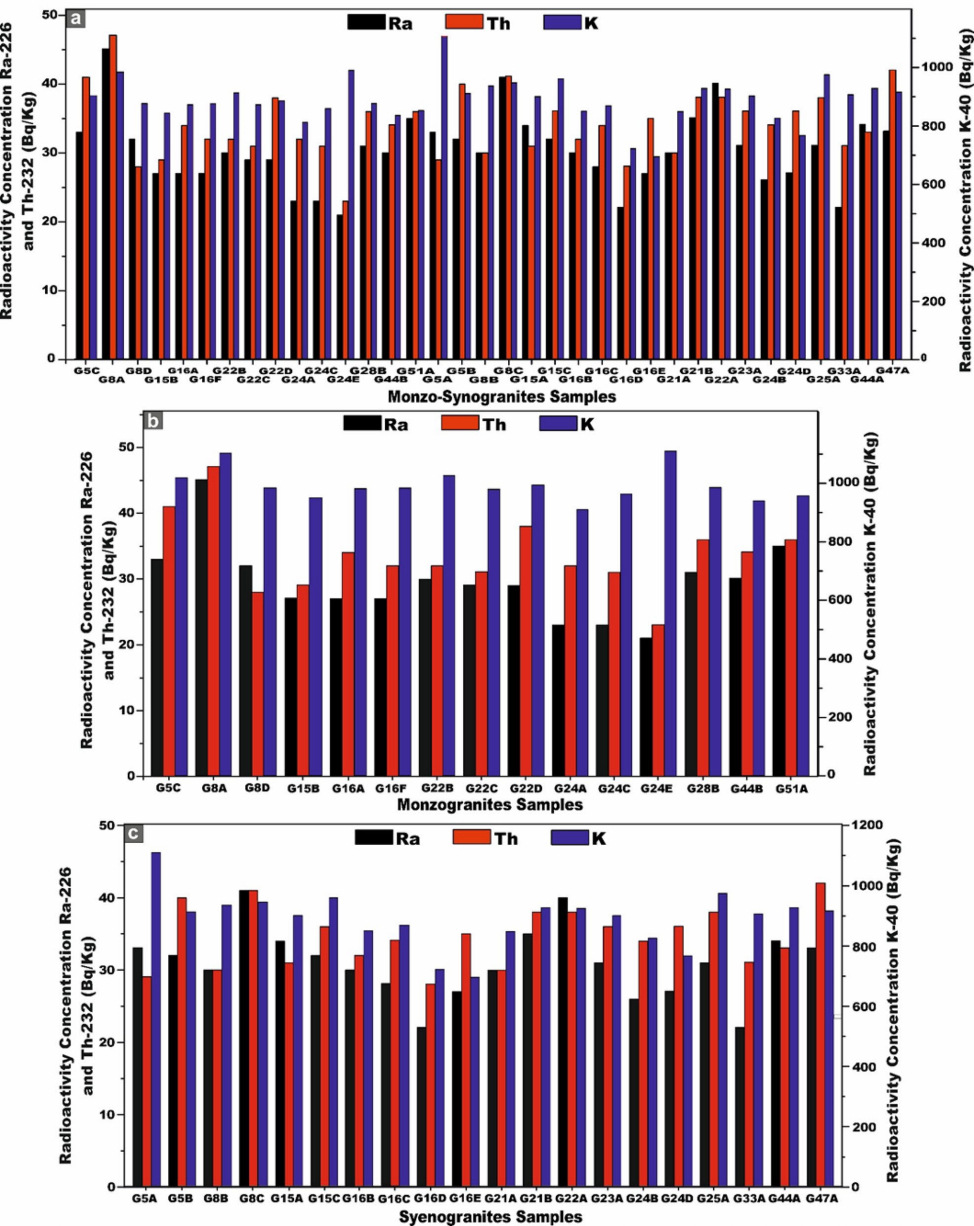
Plots of the specific activity concentrations ^226^Ra, ^232^Th, and ^40^K (Bq/kg). **(a)** for all monzo-syenogranites samples, **(b)** for monzogranites samples, and **(c)** for syenogranites samples from the Wadi El-Nabi’ area, Egyptian Nubian Shield.

Table 1 presents a comprehensive synthesis of the specific activity concentrations and statistical description of ^226^Ra (^238^U), ^232^Th, and ^40^K (in Bq/kg), together with their respective activity ratios, for all monzo-syenogranite samples examined from the Wadi El-Nabi ‘ sector of the Egyptian Nubian Shield. The pronounced variability and spatially heterogeneous distribution of radioactivity levels observed across the analyzed samples (Fig. 6a–c) likely reflect the influence of intrinsic geological heterogeneities, mineralogical controls, and post-magmatic alteration processes. These interpretations are congruent with and substantiated by the outcomes of earlier investigations^51,63^. For monzogranitoid samples, the activity concentration of ^226^Ra (^238^U) exhibits a measurable range from 21 to 45 Bq/kg, with an average value of 29 (± 6) Bq/kg. ^232^Th concentrations vary between 23 and 47 Bq/kg, yielding an average of 34 (± 6) Bq/kg, whereas ^40^K displays markedly higher levels, spanning from 811 to 989 Bq/kg, with a mean concentration of 883 (± 49) Bq/kg (Table 1). For the syenogranites samples, the activity concentration of ^226^Ra (^238^U) ranges from 22 to 41 Bq/kg, with a mean value of 31 (± 5) Bq/kg. ^232^Th concentrations extend from 28 to 42 Bq/kg, yielding an average of 35 (± 4) Bq/kg, whereas ^4^^0^K exhibits substantially elevated values, spanning from 695 to 1106 Bq/kg, with a mean concentration of 890 (± 92) Bq/kg (Table 1).

**Table 1:** Specific activity concsentrations of ^226^Ra (^238^U), ^232^Th and ^40^K and their ratios of the studied monzo-syenogranites samples from Wadi El-Nabi’ area, Egyptian Nubian Shield.

Sample code	Rock Type	C^226^Ra (^238^U) (Bq/kg)	C^232^Th (Bq/kg)	C^40^K (Bq/kg)	C^226^Ra/C^40^K	C^40^K/C^226^Ra	C^226^Ra/C^232^Th	C^232^Th/C^40^K	C^40^K/C^232^Th	C^232^Th/C^226^Ra
G5C	Monzogranite	33	41	906	0.04	27.5	0.8	0.05	22.09	1.24
G8A	Monzogranite	45	47	981	0.05	21.8	0.9	0.05	20.87	1.04
G8D	Monzogranite	32	28	875	0.04	27.34	1.14	0.03	31.25	0.88
G15B	Monzogranite	27	29	844	0.03	31.26	0.93	0.03	29.10	1.07
G16A	Monzogranite	27	34	872	0.03	32.29	0.79	0.04	25.65	1.26
G16F	Monzogranite	27	32	875	0.03	32.41	0.84	0.04	27.34	1.19
G22B	Monzogranite	30	32	912	0.03	30.4	0.94	0.04	28.5	1.07
G22C	Monzogranite	29	31	872	0.03	30.07	0.94	0.04	28.13	1.07
G22D	Monzogranite	29	38	885	0.03	30.52	0.76	0.04	23.29	1.31
G24A	Monzogranite	23	32	811	0.03	35.26	0.72	0.04	25.34	1.39
G24C	Monzogranite	23	31	858	0.03	37.30	0.74	0.04	27.67	1.35
G24E	Monzogranite	21	23	989	0.02	47.09	0.91	0.02	43	1.09
G28B	Monzogranite	31	36	877	0.04	28.29	0.86	0.04	24.36	1.16
G44B	Monzogranite	30	34	835	0.04	27.83	0.88	0.04	24.56	1.13
G51A	Monzogranite	35	36	852	0.04	24.34	0.97	0.04	26.67	1.03
G5A	Syenogranites	33	29	1107	0.03	33.54	1.14	0.03	38.17	0.88
G5B	Syenogranites	32	40	911	0.04	28.47	0.8	0.04	22.78	1.25
G8B	Syenogranites	30	30	936	0.03	31.2	1	0.03	31.2	1
G8C	Syenogranites	41	41	944	0.04	23.02	1	0.04	23.02	1
G15A	Syenogranites	34	31	900	0.04	26.47	1.09	0.03	29.03	0.91
G15C	Syenogranites	32	36	960	0.03	30	0.89	0.04	26.67	1.13
G16B	Syenogranites	30	32	850	0.04	28.33	0.94	0.04	26.56	1.07
G16C	Syenogranites	28	34	868	0.03	31	0.82	0.04	25.53	1.21
G16D	Syenogranites	22	28	721	0.03	32.77	0.79	0.04	25.75	1.27
G16E	Syenogranites	27	35	695	0.04	25.74	0.77	0.05	19.86	1.29
G21A	Syenogranites	30	30	848	0.04	28.27	1	0.04	28.27	1
G21B	Syenogranites	35	38	926	0	26.46	0.92	0.04	24.37	1.09
G22A	Syenogranites	40	38	923	0.04	23.07	1.05	0.04	24.29	0.95
G23A	Syenogranites	31	36	899	0.03	29	0.86	0.04	24.97	1.16
G24B	Syenogranites	26	34	823	0.03	31.65	0.76	0.04	24.21	1.31
G24D	Syenogranites	27	36	764	0.04	28.29	0.75	0.05	21.22	1.33
G25A	Syenogranites	31	38	972	0.03	31.35	0.82	0.04	25.58	1.23
G33A	Syenogranites	22	31	905	0.02	41.14	0.71	0.03	29.19	1.41
G44A	Syenogranites	34	33	927	0.04	27.26	1.03	0.04	28.09	0.97
G47A	Syenogranites	33	42	914	0.04	27.69	0.79	0.05	21.76	1.27
World Avg	-	35a	30a	400a	0.067b	11.43	-	-	13.33	3.49
Mean	Monzogranite	29	34	883	0.03	30.9	0.88	0.04	29.99	1.15
	Syenogranites	31	35	890	0.03	29.24	0.89	0.04	20.03	1.14
Median	Monzogranite	29	32	875	0.03	30.4	0.91	0.04	25.65	1.13
	Syenogranites	31	35	908	0.03	28.4	0.89	0.04	25.55	1.15
Standard deviation	Monzogranite	6	6	49	0.00	5.7	1	0.1	5.08	0.14
	Syenogranites	5	4	92	0.00	3.9	1.25	0.04	3.95	0.16
Minimum	Monzogranite	21	23	811	0.02	21.8	0.91	0.03	20.87	0.88
	Syenogranites	22	28	695	0.00	23.02	0.79	0.04	19.86	0.88
Maximum	Monzogranite	45	47	989	0.04	47.09	0.96	0.05	43	1.39
	Syenogranites	41	42	1106	0.04	41.14	0.98	0.04	38.17	1.41

**a** UNSCEAR. 2000^10^ **b** UNSCEAR. 1982^45^.

These results demonstrate that the mean activity concentrations of Ra (U), Th, and K in syenogranitoid samples are consistently elevated relative to their monzogranite counterparts. Moreover, the average thorium concentrations in both lithological units markedly exceed those of uranium. Owing to the exceptional resistance of Th-rich minerals to weathering, leaching and post-magmatic alteration^64^. Generally, potassium exhibits the highest activity concentration among the investigated radionuclides, a trend attributable to the abundance of K-rich feldspar minerals inherent to the granitoid formations.

Regarding the ^232^Th/^226^Ra (^238^U) ratios, monzogranite samples exhibit values ranging from 0.88 to 1.39, with an average of 1.15 (± 0.14). Syenogranite samples display a comparable range of 0.88 to 1.41, yielding an average of 1.14 (± 0.16). These ratios are markedly lower than the canonical crustal Th/Ra value of ~ 3.5^65^, indicating significant deviations from typical crustal compositions (Table 1). Such depleted Th/Ra signatures likely reflect variable degrees of magmatic differentiation, post-magmatic hydrothermal overprinting, and selective uranium enrichment within the host granitoids^66^. With respect to the ^226^Ra (^238^U)/^232^Th ratio, the monzogranite samples exhibit values ranging from 0.91 to 0.96, with a mean ratio of 0.88 (± 1). In contrast, the syenogranitoids samples display a broader variability, ranging from 0.79 to 0.98, yielding an average ratio of 0.89 (± 1.25) (Table 1). Both of the values substantially exceed the continental-crust ratio (U/Th ≈ 0.25)^67^, which strongly suggests significant uranium enrichment within these granitoid units.

The ^40^K/^226^Ra (^238^U) ratio in the monzogranite samples ranges from 21.80 to 47.09, with a mean value of 30.90 (± 5.70). In the syenogranitoids samples, this ratio varies between 23.02 and 41.14, yielding an average of 29.24 (± 3.9) (Table 1). Notably, the ratios for both granitoids types markedly exceed the globally reported average value of 11.43^10^ (Table 1), underscoring a pronounced enrichment in ^40^K relative to ^226^Ra (^238^U). Finally, the ^40^K/^232^Th ratios exhibit considerable variability, ranging from 20.87 to 43.00 with a mean value of 26.99 (± 5.08) in the monzogranitoids samples, and from 19.86 to 38.17 with an average of 20.03 (± 3.95) in the syenogranitoids rocks (Table 1). Notably, the ratio values in both lithological units substantially exceed the globally recognized average of 13.33^10^, indicating a pronounced relative enrichment in ^40^K relative to ^232^Th.

**Fig. 7:**
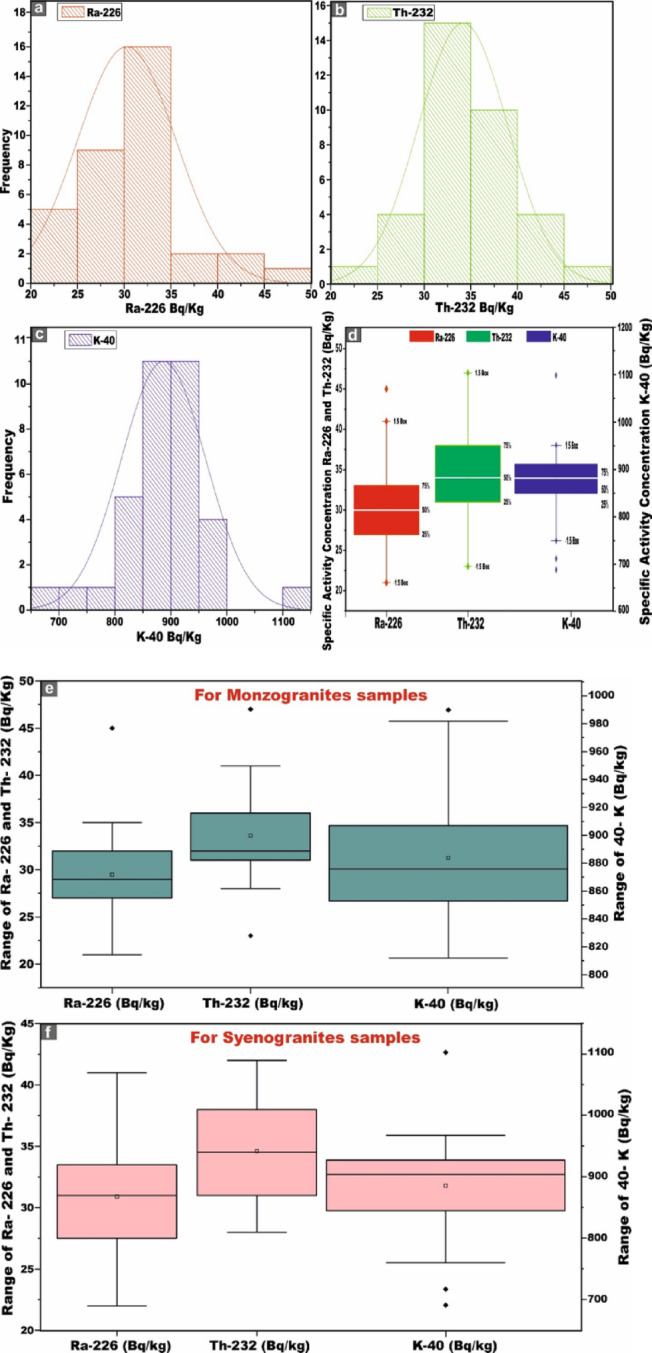
**(a, b, c)** Distribution frequency histograms curves of ^226^Ra, ^232^Th, and ^40^K (Bq/kg) activity concentrations for all monzo-syenogranites samples, respectively. **(d)** The box diagram shows the basic statistics of the distribution of radionuclides (^226^Ra, ^232^Th, and ^40^K Bq/kg) for all monzo-syenogranite samples. **(e)** box diagram shows the basic statistics of the distribution of radionuclides (^226^Ra, ^232^Th and ^40^K Bq/kg) for monzogranites samples, and **(f)** box diagram shows the basic statistics of the distribution of radionuclides (^226^Ra, ^232^Th and ^40^K Bq/kg) for syenogranites samples from Wadi El-Nabi’ area, Egyptian Nubian Shield.

Skewness is a frequency distribution that refers to the degree of asymmetry observed around its central tendency. A distribution is considered skewed when it deviates from perfect symmetry, manifesting either a positive (right-tailed) or negative (left-tailed) inclination. Figure 7 provides an insightful overview of the frequency distribution of specific activity concentrations of radionuclides ^226^Ra (^238^U), ^232^Th, and ^40^K (Bq/kg) across the entire 35 monzo-syenogranite samples collected from the study area. Notably, the ^226^Ra (^238^U) histogram shows a moderately right-skewed distribution. The fitted curve confirms that the dataset approximates a unimodal distribution with a slight tail toward elevated concentrations (Fig. 7a). While the ^232^Th histogram displays a near-normal distribution (symmetric bell-shaped), reflecting a relatively homogeneous thorium content across the samples (Fig. 7b). ^40^K exhibits a broad, symmetric spectrum, indicating a well-balanced distribution, influenced primarily by the mineralogical composition of feldspar-bearing phases (Fig. 7c).

Figure 7d enhances the visualization of the principal descriptive statistical parameters for the analyzed radionuclides across all monzo-syenogranites samples through boxplots, the corresponding numerical values of which are systematically consolidated in Table 1. Figure 7e, f further delineate the data, providing separate boxplot representations for monzogranites and syenogranites, respectively, thereby facilitating a more granular comparative analysis.

The mean concentrations of the radioisotopes ^226^Ra (^238^U), ^232^Th, and ^40^K in the monzogranite samples are 29, 34, and 883 Bq/kg, respectively. In contrast, the syenogranites samples exhibit marginally higher mean values of 31, 35, and 890 Bq/kg for ^226^Ra (^238^U), ^232^Th, and ^40^K, respectively. Notably, the concentrations in syenogranites consistently exceed those observed in monzogranites. To enable a rigorous and comprehensive assessment of the mean specific activity concentrations of radionuclides in the analyzed granitic lithologies relative to internationally reported benchmark values, multiple reference datasets published by UNSCEAR across different reporting periods were utilized. These include UNSCEAR 1993 averages (^22^⁶Ra (^238^U) = 50 Bq/kg; ^232^Th = 50 Bq/kg; ^4^⁰K = 500 Bq/kg)^68^, the 2000 dataset (^226^Ra (^238^U) = 35 Bq/kg; ^232^Th = 30 Bq/kg; ^40^K = 400 Bq/kg)^10^, and the revised 2008 values (^226^Ra (^238^U) = 370 Bq/kg; ^232^Th = 45 Bq/kg; ^40^K = 412 Bq/kg)^69^. Additionally, the UNSCEAR 2010 publication provides further consolidated global averages, reporting (^226^Ra (^238^U) = 32 Bq/kg; ^232^Th = 45 Bq/kg; and ^40^K = 412 Bq/kg)^14^.

**Fig. 8:**
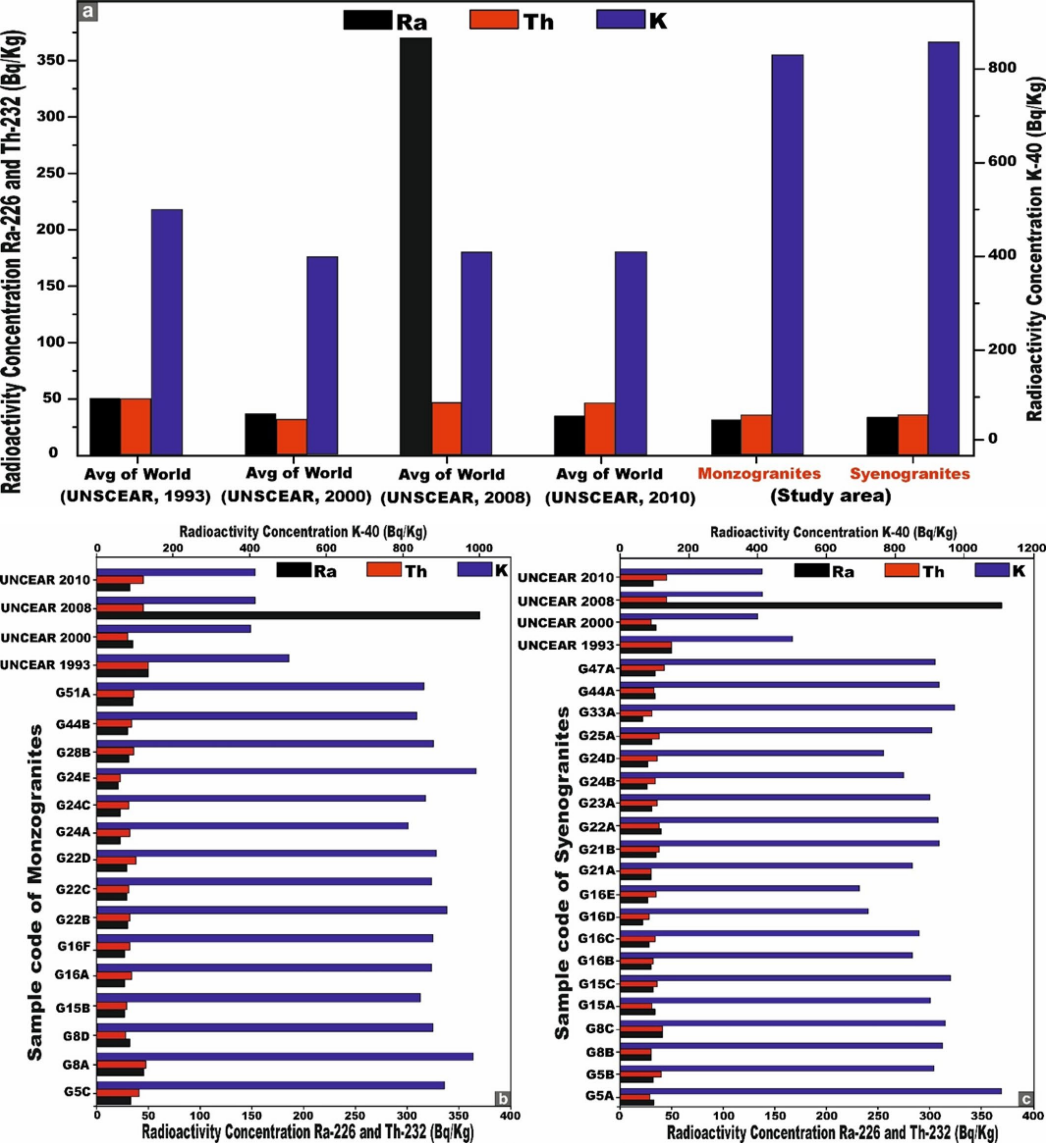
**(a)** Comparison between specific activity concentrations ^226^Ra, ^232^Th, and ^40^K of monzogranites and syenogranites in our study and the world average concentrations of granitic rocks as referred to (UNSCEAR, 1993, 2000, 2008, and 2010). UNSCEAR, 1993^67^ values (^226^Ra = 50, ^232^Th = 50 and ^40^K = 500 Bq/kg), UNSCEAR, 2000^10^ values (^226^Ra = 35, ^232^Th = 30 and ^40^K = 400 Bq/kg), UNSCEAR, 2008^68^ values (^226^Ra = 370, ^232^Th = 45 and ^40^K = 412 Bq/kg), UNSCEAR, 2010^14^ values (^226^Ra = 32, ^232^Th = 45 and ^40^K = 412 Bq/kg). Comparison between specific activity concentrations ^226^Ra, ^232^Th, and ^40^K. **(b)** monzogranites, and **(c)** for syenogranites in our study and the world average concentrations of granitic rocks as referred to (UNSCEAR, 1993, 2000, 2008, and 2010). UNSCEAR, 1993^67^ values (^226^Ra = 50, ^232^Th = 50 and ^40^K = 500 Bq/kg), UNSCEAR, 2000^10^ values (^226^Ra = 35, ^232^Th = 30 and ^40^K = 400 Bq/kg), UNSCEAR, 2008^68^ values (^226^Ra = 370, ^232^Th = 45 and ^40^K = 412 Bq/kg), UNSCEAR, 2010^14^ values (^226^Ra = 32, ^232^Th = 45 and ^40^K = 412 Bq/kg).

For ^40K^, the measured concentrations surpass all corresponding global reference values reported by UNSCEAR in 1993, 2000, 2008, and 2010. In the case of ^232^Th, the obtained values exceed the UNSCEAR 2000 global average, yet remain below the benchmark levels documented in the 1993, 2008, and 2010 reports (Fig. 8). Conversely, for ^226^Ra (^238^U), the measured concentrations are consistently lower than the worldwide reference values provided by UNSCEAR across all reporting periods (1993, 2000, 2008, and 2010) (Fig. 8).

### Variation diagrams

**Fig. 9:**
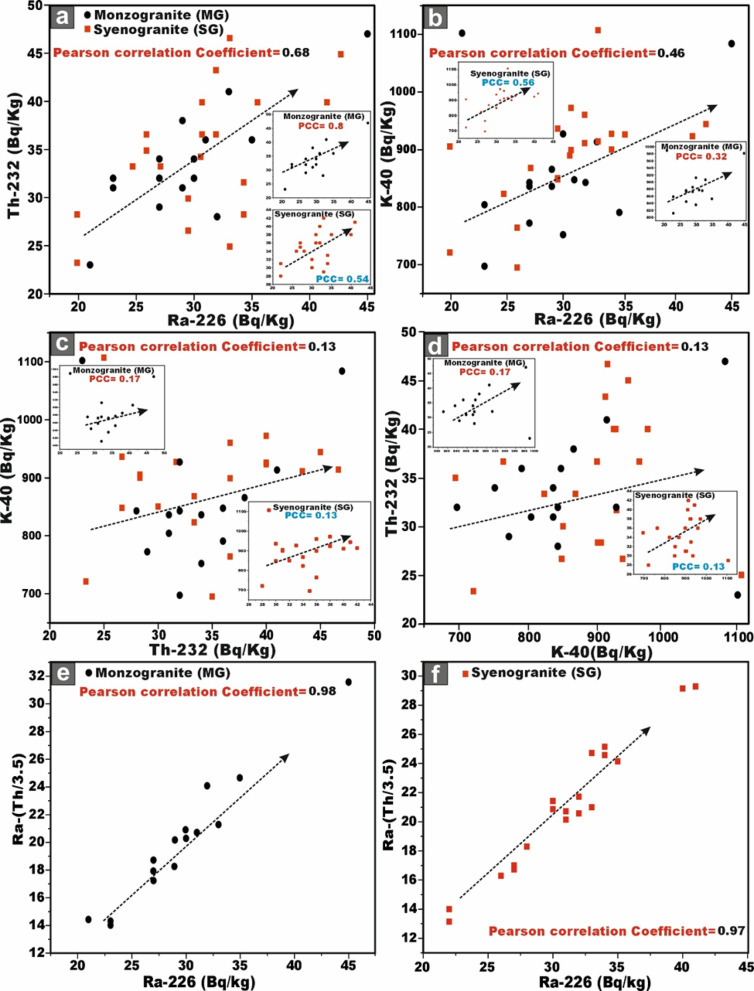
**(a-d)** Variation diagrams between **(a-b)** ^226^Ra (Bq/kg) vs. ^232^Th and ^40^K (Bq/kg), **(c)** ^232^Th and ^40^K (Bq/kg), **(d)** ^40^K and ^232^Th (Bq/kg) for the studied monzo-syenogranitoids samples, and **(e, f)** ^226^Ra (Bq/kg) vs. ^226^Ra- (^232^Th/3.5) for monzogranite and syenogranite samples.

This analytical approach provides a robust statistical framework for assessing the degree of linear association between the studied radionuclides. The integrated evaluation of the relationship between ^226^Ra (^238^U) and ^232^Th across all granitoids samples reveals a moderately positive correlation (r = 0.68) (Fig. 9a). However, when the datasets are partitioned by lithological type, a strong positive correlation between ^226^Ra (^238^U) and ^232^Th emerges within the monzogranite samples (r = 0.8), this means that the existence of the Ra is dependent on the presence of Th (Fig. 9a). Whereas the syenogranites assemblage exhibits only a moderately positive correlation (r = 0.54) (Fig. 9a). This posotive correlations underscores the magmatic origin of the radionuclides ^226^Ra and ^232^Th ^70^, as well as the coexistence of these radionuclide elements in radioactive accessory minerals such as zircon, xenotime, and thorite.

With respect to the relation between ^226^Ra (^238^U) and ^40^K across all investigated granite samples, the correlation is characterized as weakly positive (r = 0.46) (Fig. 9b). However, upon differentiating the lithological types, a weak positive correlation persists within the monzogranites samples (r = 0.32) (Fig. 9b), whereas the syenogranites assemblage exhibits a moderately positive correlation (r = 0.56) (Fig. 9b).

When evaluating the interrelationship between ^232^Th and ^40^K in all investigated granite samples, the correlation is discerned to be weakly positive (r = 0.13) (Fig. 9c, d). Likewise, upon segregating the granitoid types, the same weak positive association persists in both directions of the relationship (r = 0.17 and 0.13), respectively for monzogranites and syeenogranites samples (Fig. 9c, d), indicating ^40^K is widely distributed as a primary component in various types of rocks, including sedimentary, magmatic, and metamorphic formations.

Furthermore, the relationship between ^226^Ra and [Ra- (Th/3.5)] parameter exhibits an exceptionally strong positive correlation in both monzogranitoids and syenogranitoids samples, with correlation coefficients of 0.98 and 0.97, respectively (Fig. 9e, f). This pronounced linear association is highly indicative of Ra (U) enrichment and subsequent mobilization processes facilitated by hydrothermal fluid activity. Additional support for this interpretation arises from the markedly elevated average values of [Ra- (Th/3.5)], calculated as 19.87 > 0 for the monzogranites and 21.01 > 0 for the syenogranites.

These relationships were validated not only through the computation of Pearson correlation coefficients (r) but also through the graphical representation of the best-fit line using Origin software.

### c. Radiological effects

Tables 2 and 3 summarize the radiological health-hazard indices for the analyzed monzogranites and syenogranites samples from the Wadi El-Nabi’ region, situated within the Egyptian Nubian Shield.

**Table 2:** Radiological health hazard indices for the studied monzo-syenogranites from Wadi El-Nabi’, Egyptian Nubian Shield.

Sample code	Rock Type	Ra_eq_ (Bq/kg)	D(nGy/h)		AUI	AEDE (mSv/year)		H_ex_	H_in_	ELCR_out_ × 10^–3^	ELCR_in_ × 10^–3^	I_γ_	I_α_	AGDE μSv y ^− 1^
			D_out_ (nGy/h)	D_in_ (nGy/h)		Out door	In door							
G5C	MG	162	77	150	0.9	0.09	0.73	0.44	0.53	0.33	2.57	1.23	0.2	557.8
G8A	MG	187	89	174	1.1	0.11	0.85	0.51	0.63	0.38	2.98	1.42	0.2	643.5
G8D	MG	140	67	132	0.7	0.08	0.65	0.38	0.47	0.29	2.27	1.08	0.2	490. 7
G15B	MG	134	64	126	0.7	0.08	0.62	0.36	0.44	0.28	2.16	1.03	0.1	469. 7
G16A	MG	144	69	134	0.7	0.08	0.66	0.39	0.46	0.29	2.30	1.1	0.1	499.4
G16F	MG	140	67	132	0.7	0.08	0.65	0.38	0.45	0.29	2.26	1.08	0.1	491.9
G22B	MG	146	70	138	0.7	0.09	0.67	0.39	0.47	0.30	2.36	1.13	0.2	512.8
G22C	MG	140	68	132	0.7	0.08	0.65	0.38	0.46	0.29	2.27	1.08	0.1	492. 9
G22D	MG	151	72	141	0.8	0.09	0.69	0.41	0.49	0.31	2.42	1.16	0.1	526.3
G24A	MG	131	63	123	0.7	0.08	0.6	0.35	0.41	0.27	2.11	1.01	0.1	459.5
G24C	MG	134	64	126	0.7	0.08	0.62	0.36	0.43	0.28	2.16	1.04	0.1	470.1
G24E	MG	130	64	126	0.6	0.08	0.62	0.35	0.41	0.28	2.16	1.03	0.1	471.6
G28B	MG	150	72	140	0.8	0.09	0.69	0.41	0.49	0.31	2.40	1.15	0.2	521.7
G44B	MG	143	68	133	0.8	0.08	0.65	0.39	0.47	0.29	2.29	1.09	0.2	497
G51A	MG	153	72	142	0.8	0.09	0.69	0.41	0.51	0.31	2.43	1.16	0.2	526.2
G5A	SG	160	78	153	0.7	0.1	0.75	0.43	0.52	0.33	2.63	1.25	0.2	570.8
G5B	SG	159	76	148	0.9	0.09	0.73	0.43	0.52	0.33	2.54	1.22	0.2	522
G8B	SG	145	70	137	0.7	0.09	0.67	0.39	0.47	0.30	2.36	1.12	0.2	512
G8C	SG	172	82	160	1	0.1	0.79	0.46	0.58	0.35	2.75	1.31	0.2	594.5
G15A	SG	148	71	139	0.8	0.09	0.68	0.4	0.49	0.30	2.39	1.14	0.2	517
G15C	SG	157	76	148	0.8	0.09	0.72	0.42	0.51	0.32	2.54	1.21	0.2	551
G16B	SG	142	68	133	0.7	0.08	0.65	0.38	0.47	0.29	2.27	1.09	0.2	493.4
G16C	SG	143	69	134	0.7	0.08	0.66	0.39	0.46	0.30	2.31	1.1	0.1	501.2
G16D	SG	117	56	110	0.6	0.07	0.54	0.32	0.38	0.24	1.89	0.91	0.1	411.4
G16E	SG	130	62	120	0.7	0.08	0.59	0.35	0.43	0.26	2.07	0.99	0.1	448
G21A	SG	139	66	130	0.7	0.08	0.64	0.38	0.46	0.29	2.23	1.07	0.2	484.4
G21B	SG	161	77	150	0.9	0.09	0.74	0.43	0.53	0.33	2.57	1.23	0.2	557.8
G22A	SG	166	79	154	0.9	0.1	0.76	0.45	0.56	0.34	2.65	1.26	0.2	572
G23A	SG	152	73	142	0.8	0.09	0.7	0.41	0.49	0.31	2.44	1.17	0.2	528.6
G24B	SG	138	66	129	0.7	0.08	0.63	0.37	0.44	0.28	2.21	1.06	0.1	480.9
G24D	SG	137	65	127	0.7	0.08	0.62	0.37	0.44	0.28	2.18	1.05	0.1	473.8
G25A	SG	159	77	150	0.8	0.09	0.74	0.43	0.51	0.33	2.58	1.23	0.2	559.8
G33A	SG	135	66	129	0.7	0.08	0.63	0.37	0.43	0.28	2.21	1.06	0.1	481.7
G44A	SG	152	73	68	0.8	0.09	0.7	0.41	0.5	0.27	2.31	1.17	0.2	534.1
G47A	SG	163	78	77	0.9	0.1	0.74	0.44	0.53	0.28	2.4	1.25	0.2	564.5
Mean (Avg.)	MG	146	70	137	0.8	0.09	0.67	0.39	0.47	0.3	2.34	1.12	0.1	508.74
	SG	149	71	132	0.8	0.09	0.68	0.40	0.49	0.3	2.38	1.15	0.2	519.4
World Avg	–-	370	54	85		0.48	0.48	1	1	0.29*10^–3^	1.16*10^–3^	1	1	300
Min	MG	130	63	123	0.6	0.08	0.6	0.35	0.41	0.27	2.11	1.01	0.1	459.5
	SG	117	56	67.6	0.6	0.07	0.54	0.32	0.38	0.24	1.89	0.91	0.1	411
Max	MG	187	89	174	1.1	0.11	0.85	0.51	0.63	0.38	2.98	1.42	0.2	643.5
	SG	172	82	160	1	0.1	0.79	0.46	0.58	0.35	2.75	1.31	0.2	594
SD	MG	14.4	6.6	13	0.1	0.01	0.06	0.41	0.05	0.03	0.22	0.1	0.05	45.7
	SG	13.8	6.7	24	0.1	0.01	0.06	0.04	0.05	0.03	0.22	0.10	0.05	47.34

**MG = Monzogranite SG = Syenogranite.**

**Table 3:** Calculation of the effective dose rate delivered to the organs (D_organ_) for the studied monzo-syenogranites from Wadi El-Nabi’, Egyptian Nubian Shield.

Sample code	Rock Type	AEDE total	D (lungs)	D (ovaries)	D (Bone Marrow)	D (Testes)	D (Entire body)
G5C	Monzogranite	0.82	0.52	0.48	0.57	0.67	0.56
G8A	Monzogranite	0.96	0.61	0.56	0.66	0.79	0.65
G8D	Monzogranite	0.73	0.47	0.42	0.50	0.59	0.49
G15B	Monzogranite	0.7	0.45	0.41	0.48	0.57	0.48
G16A	Monzogranite	0.74	0.47	0.43	0.51	0.61	0.50
G16F	Monzogranite	0.73	0.47	0.42	0.50	0.59	0.49
G22B	Monzogranite	0.76	0.49	0.44	0.52	0.62	0.52
G22C	Monzogranite	0.73	0.47	0.42	0.50	0.59	0.49
G22D	Monzogranite	0.78	0.49	0.45	0.54	0.64	0.53
G24A	Monzogranite	0.68	0.44	0.39	0.47	0.56	0.46
G24C	Monzogranite	0.7	0.45	0.41	0.48	0.57	0.48
G24E	Monzogranite	0.7	0.45	0.41	0.48	0.57	0.48
G28B	Monzogranite	0.78	0.49	0.45	0.54	0.64	0.53
G44B	Monzogranite	0.73	0.47	0.42	0.50	0.59	0.49
G51A	Monzogranite	0.78	0.49	0.45	0.54	0.64	0.63
G5A	Syenogranite	0.85	0.54	0.49	0.59	0.69	0.58
G5B	Syenogranite	0.82	0.52	0.48	0.57	0.67	0.56
G8B	Syenogranite	0.76	0.49	0.44	0.52	0.62	0.52
G8C	Syenogranite	0.89	0.57	0.52	0.61	0.73	0.61
G15A	Syenogranite	0.77	0.49	0.45	0.53	0.63	0.52
G15C	Syenogranite	0.81	0.52	0.47	0.56	0.66	0.55
G16B	Syenogranite	0.73	0.47	0.42	0.50	0.59	0.49
G16C	Syenogranite	0.74	0.47	0.43	0.51	0.61	0.50
G16D	Syenogranite	0.61	0.39	0.35	0.42	0.5	0.41
G16E	Syenogranite	0.67	0.43	0.39	0.46	0.55	0.56
G21A	Syenogranite	0.72	0.46	0.42	0.49	0.59	0.49
G21B	Syenogranite	0.83	0.53	0.48	0.57	0.68	0.56
G22A	Syenogranite	0.86	0.55	0.49	0.59	0.71	0.58
G23A	Syenogranite	0.79	0.51	0.46	0.55	0.65	0.54
G24B	Syenogranite	0.71	0.45	0.41	0.49	0.58	0.48
G24D	Syenogranite	0.7	0.45	0.41	0.48	0.57	0.48
G25A	Syenogranite	0.83	0.53	0.48	0.57	0.68	0.56
G33A	Syenogranite	0.71	0.45	0.41	0.49	0.58	0.48
G44A	Syenogranite	0.79	0.51	0.46	0.55	0.65	0.54
G47A	Syenogranite	0.84	0.53	0.49	0.57	0.69	0.57
Mean	Monzogranite	0.75	0.48	0.44	0.52	0.62	0.51
	Syenogranite	0.77	0.49	0.45	0.53	0.63	0.52

**Fig. 10:**
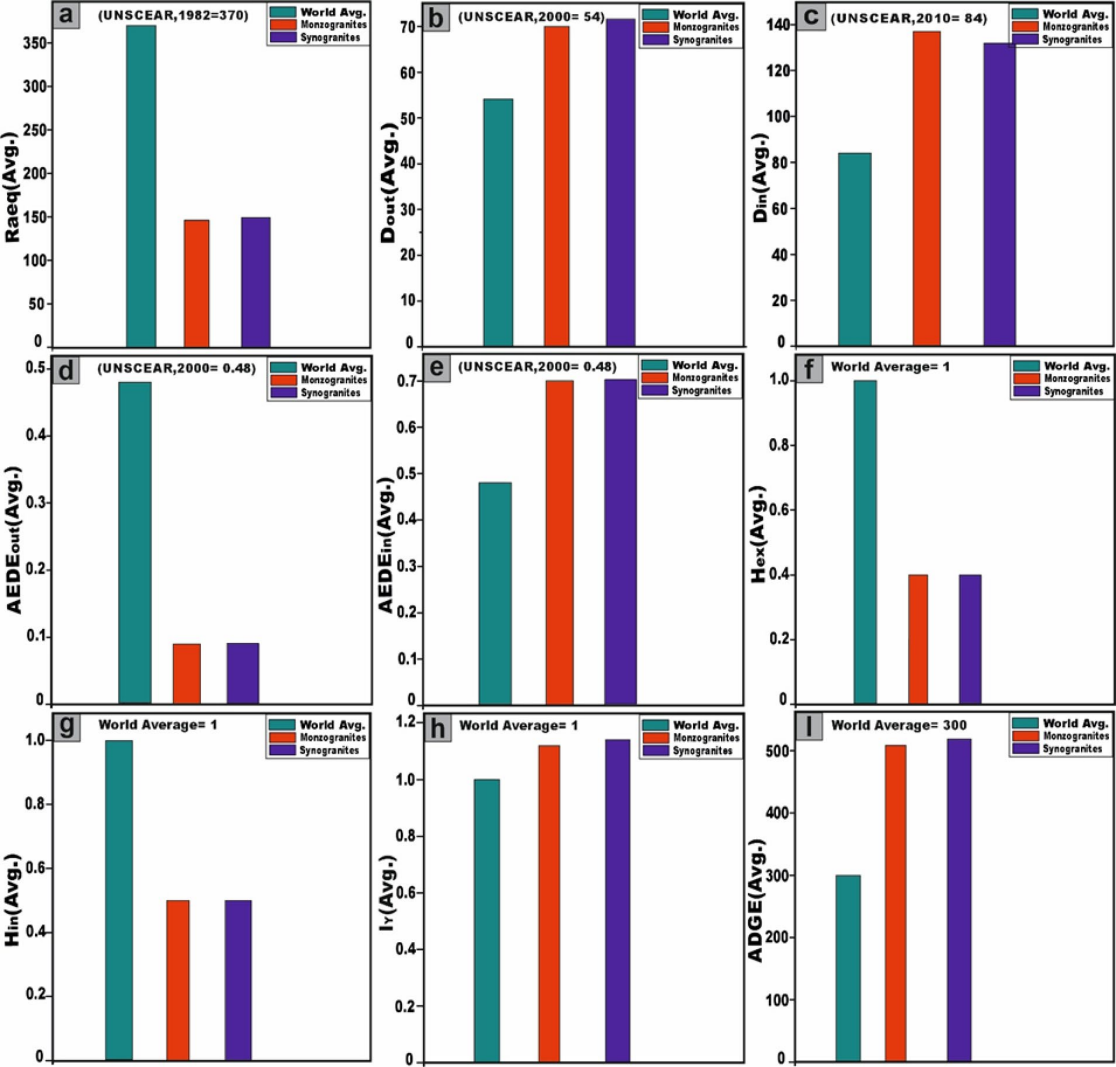
**(a-i)** Comparative assessment of radiological hazard index values for the studied monzogranite and syenogranite lithologies (Average values) relative to internationally established benchmark values.

The radium equivalent activity (Ra_eq_) values for the monzogranite samples range from 130 to 187 Bq/kg, with a mean value of 146 (± 14.4) Bq/kg. Conversely, the syenogranites samples display Ra_eq_ values spanning from 117 to 172 Bq/kg, with an average of 149 (± 13.8) Bq/kg (Table 2). Evidently, all recorded values fall well below the recommended safety threshold of 370 Bq/kg, as specified by^45^ (Fig. 10a). Thus, all the granitoids types might be safe building materials with no significant radiological hazard.

**Fig. 11:**
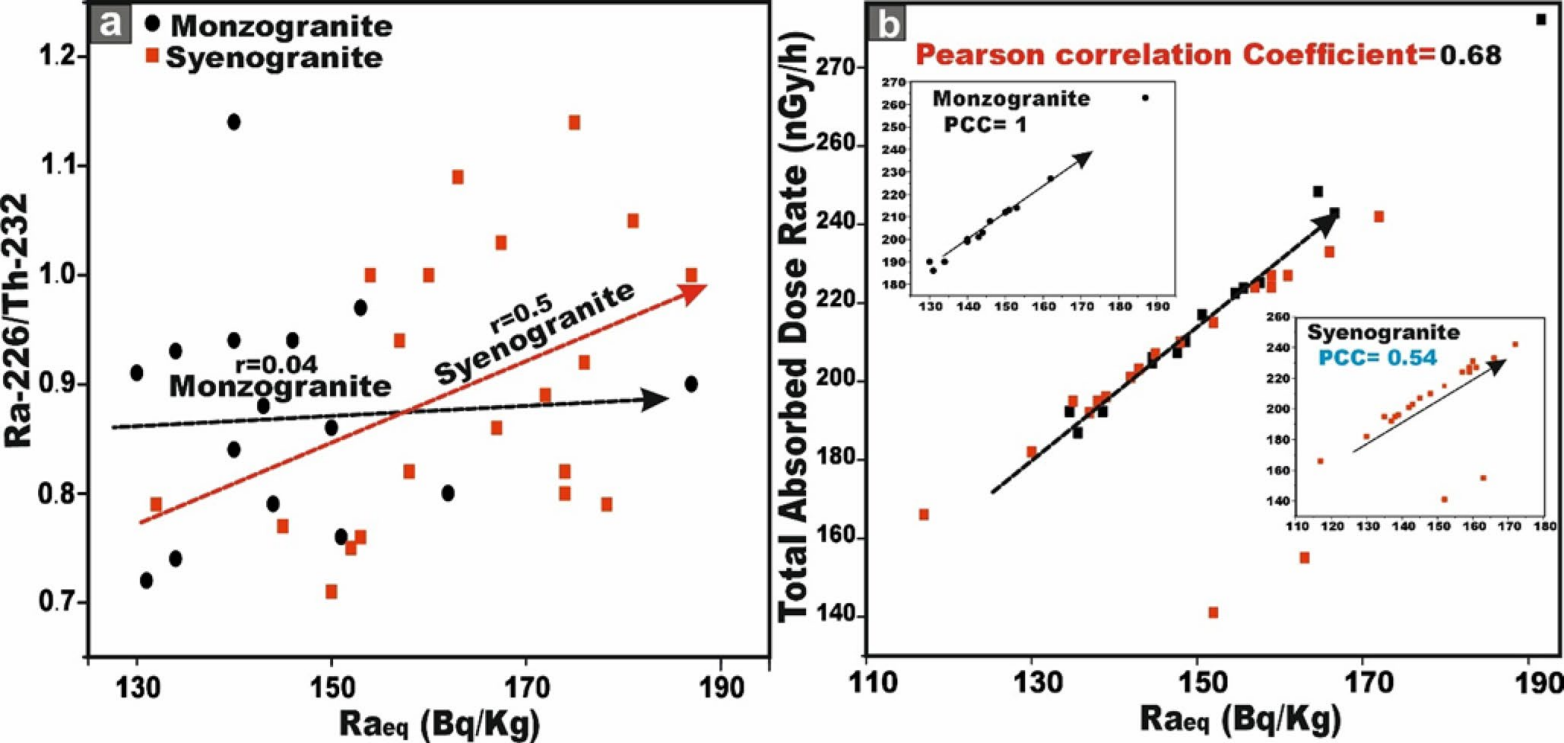
**(a)** Correlation between Ra_eq_ (Bq/kg) and ^226^Ra/^232^Th, and **(b)** correlation between Ra_eq_ (Bq/kg) and absorbed dose rate (D) for the studied monzogranite and syenogranite samples.

Regarding the relationship between Ra_eq_ (Bq/kg) and the ^226^Ra/^232^Th ratio in the granitoids samples, the monzogranites exhibit a very weak positive correlation (r = 0.04), whereas the syenogranite samples display a moderate positive correlation (r = 0.5) (Fig. 11a).

The gamma absorbed dose rate in the air (D) was evaluated, revealing outdoor values (D_out_) ranging from 63 to 89 nGy/h with an average of 70 (± 6.6) nGy/h and from 56 to 82 nGy/h with an average of 71 (± 6.7) nGy/h for monzogranites and syenogranites, respectively. It is important to highlight these measurements because they exceed the safety thresholds of 54 nGy/h, as advised by^10^ (Fig. 10b). Moreover, the indoor values (D_in_) for the same samples, were ranging from 123 to 174 nGy/h and from 67.6 to 160 nGy/h with an average of 137 (± 13) and 132 (± 24) nGy/h for monzogranites and syenogranites, respectively (Table 2), surpassing the safety limit of 84 nGy/h, according to^10^ (Fig. 10c). This finding indicates that the granitic rocks within the study area are unsuitable for use in construction or any other infrastructural applications.

**Fig. 12:**
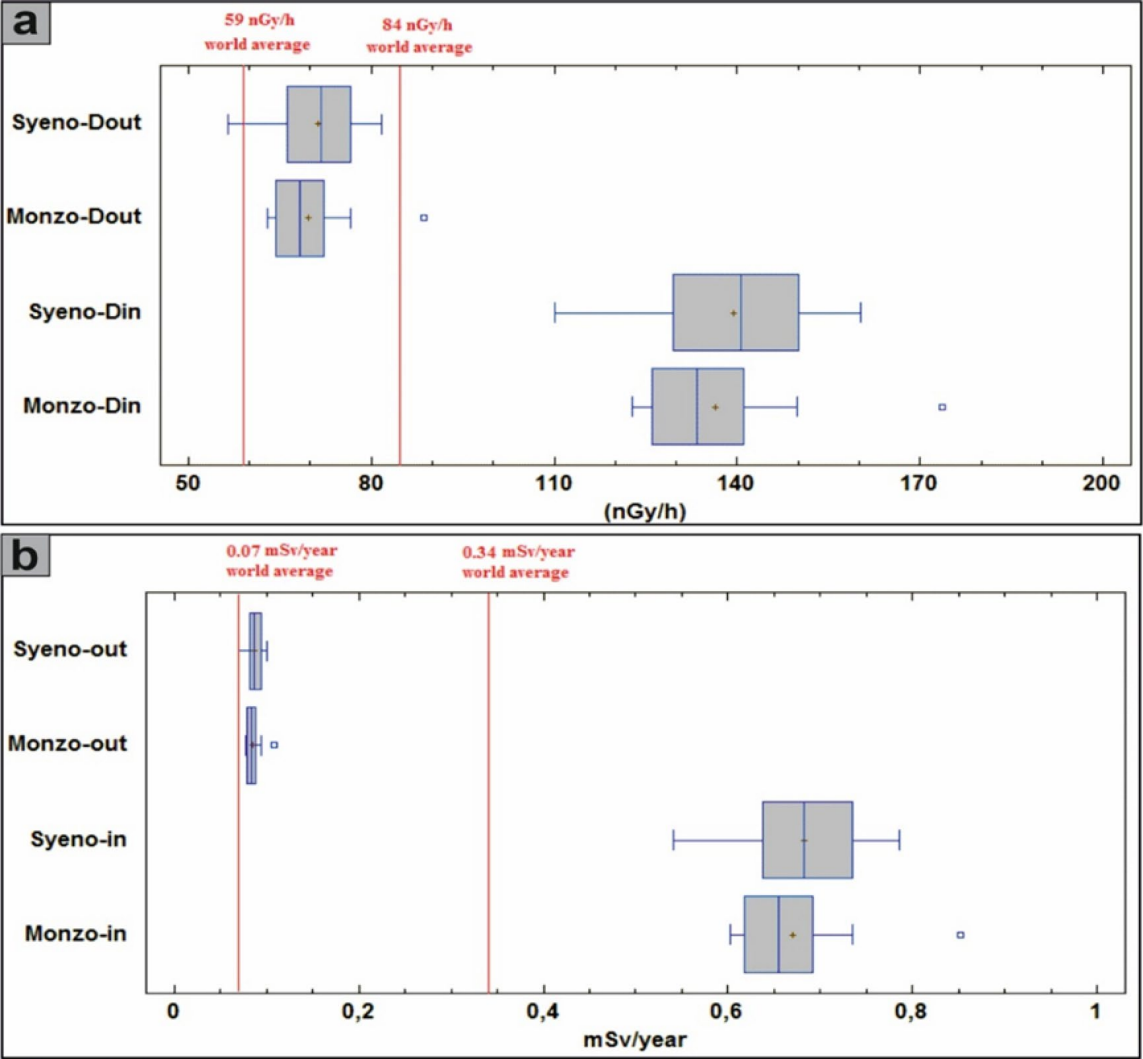
**(A)** Distributions of D_out_ and D_in_ values for comparison using box and whisker plots. Red lines indicate the world average, and **(b)** the comparison of the AEDE_in_ and AEDE_out_ values of the samples with the help of box and whisker plots. Abbreviations Syeno-in and Monzo-in are AEDE_in_ values for syenogranites and monzogranites, respectively, and Syeno-out and Monzo-out are AEDE_out_ values for syenogranites and monzogranites, respectively.

The relationship between ^226^Ra (Bq/kg) and total gamma absorbed dose rate (D_in_ + D_out_), exhibits a moderately positive correlation (r = 0.68), when all samples are considered collectively. However, upon separating the lithologies, the monzogranites display a strong positive correlation (r = 1), whereas the syenogranites demonstrate a moderate positive correlation (r = 0.54) (Fig. 12a). This behavior underscores the dominant influence of ^226^Ra (^238^U) on the gamma-ray emission budget of the rocks. The distributions of D_out_ and D_in_ are further illustrated for comparative assessment using box and whisker plots (Fig. 12a).

The analysis of the Activity Utilization Index (AUI) revealed values ranging from 0.6 to 1.1 Bq/kg for monzogranites and from 0.6 to 1.0 Bq/kg for syenogranites, with average values of 0.8 (± 0.1) Bq/kg for both rock types (Table 2). Notably, the majority of these values remain below the recommended safety threshold of 1. However, two exceptions were identified: samples G8A (monzogranite) and G8C (syenogranite), which marginally exceed the permissible limit.

Concerning the annual effective dose equivalent (AEDE), the outdoor component (AEDE_out_) exhibits values ranging from 0.08 to 0.11 mSv/y for the monzogranitic samples and from 0.07 to 0.10 mSv/y for the syenogranitic counterparts, yielding comparable mean values of approximately 0.09 (± 0.01) mSv·y^-1^ for both lithological units (Table 2). It is noteworthy that all recorded values fall well below the internationally recommended safety threshold of 0.48 mSv/y^10^ for AEDE_out_ (Fig. 10d). Concurrently, the indoor values (AEDE_in_) varied from 0.60 to 0.85 mSv/y for monzogranites and 0.54 to 0.79 mSv/y for syenogranites, yielding average values of 0.67 (± 0.06) and 0.68 (± 0.06) mSv/y, respectively (Table 2). It is worth noting that these values surpass the safety limit of 0.48 mSv/year^10^ for AEDE_in_ (Fig. 10e). The comparison of AEDE_in_ and AEDE_out_ values for the samples is conducted using box and whisker plots (Fig. 12b).

**Fig. 13:**
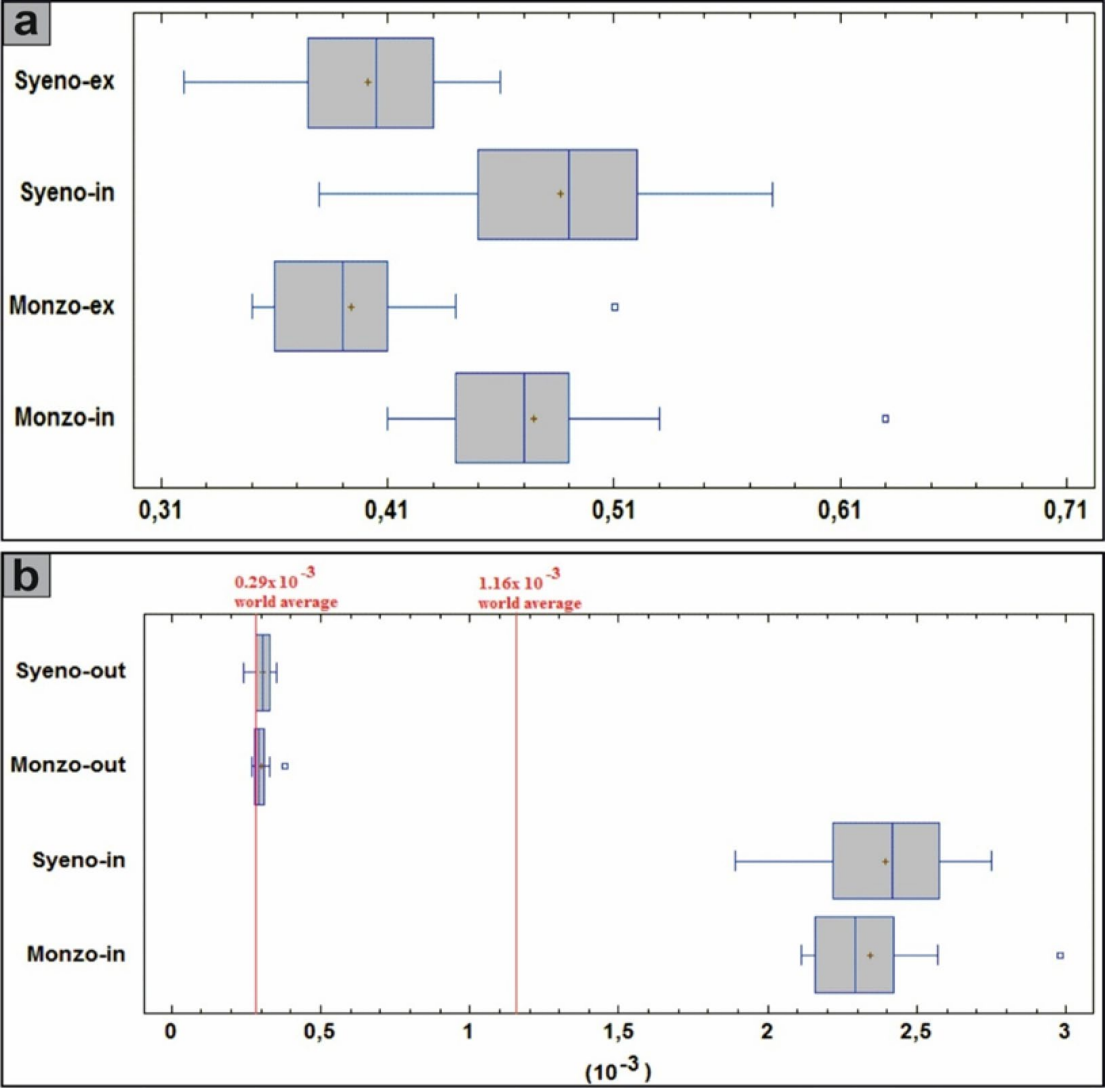
**(a)** The comparison of H_in_ and H_ex_ values of the samples with the help of box and whisker plots. abbreviations Syeno-ex and Monzo-ex are H_ex_ values of syenogranites and monzogranites, respectively. Additionally, Syeno-in and Monzo-in are the abbreviations of H_in_ values for syenogranites and monzogranites, respectively, and **(b)** comparison of ELCR_in_ and ELCR_out_ values for the studied monzogranites and syenogranites with the help of box and whisker plots.

Of the external and internal hazard indices (H_ex_ and H_in_), and given that a substantial proportion of adults dedicate approximately 80% of their time indoors, it becomes evident that indoor radiation sources contribute significantly to their overall radiation exposure compared to outdoor sources^71^ because of gamma-rays, radon gas, and its decay products. The calculated values of the (H_ex_) ranged from 0.35 to 0.51 for monzogranite samples and 0.32 to 0.46 for syenogranite samples, with average values of 0.39 (± 0.41) and 0.40 (± 0.04) for granitic samples, respectively (Table 2). Additionally, the internal values (H_in_) for granitic samples ranged from 0.41 to 0.63 and from 0.38 to 0.58 with an average of 0.47 (± 0.05) and 0.49 (± 0.05) for monzogranite and syenogranite samples, respectively (Table 2). Notably, these findings (H_ex_ and H_in_) fall below the prescribed safety limit of < 1 (unity), as recommended by^51,52^ (Fig. 10f, g). This reflects that there is no significant risk associated with these granitic rocks and negligible hazard effects of radon and its short-lived progeny on the respiratory organs^10^. Utilizing box and whisker plots, the H_in_ and H_ex_ values of the samples are compared (Fig. 13a).

With respect to the excess lifetime cancer risk (ELCR), the outdoor Excess lifetime cancer risk (ELCR_out_) values ranged from 0.27 to 0.38 mSv/y, with a mean value of 0.30 (± 0.03) mSv/y for monzogranites. Similarly, for syenogranites, the values ranged from 0.24 to 0.35 mSv/y, also yielding an average of 0.30 (± 0.03) mSv/y (Table 2). Simultaneously, the indoor Excess Lifetime Cancer Risk (ELCR_in_) values ranged from 2.11 to 2.98 for monzogranites and from 1.89 to 2.75 for syenogranites, with average values of 2.34 (± 0.22) and 2.38 (± 0.22), respectively (Table 2). Notably, these ELCR_out_ and ELCR_in_ results fall higher than and do not agree with the accessible average value of 0.29 × 10^−3^^10^, which produce health risks and may cause cancers and other serious diseases. The comparison of ELCR_in_ and ELCR_out_ values for the investigated monzo-syenogranites is conducted through the utilization of box and whisker plots (Fig. 13b).

Regarding the representative gamma index (I_γ_), the results revealed values ranging from 1.01 to 1.42 for monzogranites and 0.91 to 1.31 for syenogranites, with average values of 1.12 (± 0.1) and 1.15 (± 0.1), respectively (Table 2). Notably, these values exceed the recommended threshold and the dose criterion of 1 mSv/y (unity) (Fig. 10h), indicating a heightened radiological concern. For the alpha index (I_α_), the mean values were determined to be 0.1 (± 0.05) and 0.2 (± 0.05) for the monzogranites and syenogranites samples, respectively (Table 2). It is noteworthy that these values remain well below the recommended safety threshold of 0.5 Bq/kg, thereby indicating compliance with established radiological safety standards.

For the Annual Gonadal Dose Equivalent (AGDE), the mean values for AGDE (μSv/y) are outlined in Table 2 and visualized in Fig. 10i. The recorded average values are 509 (± 45.7) and 519 (± 47.34) μSv/y for the monzogranites and syenogranites samples, respectively, exceeding the global average of 300 μSv/y^72,73^. This clearly underscores a significant radiological risk associated with these particular granitic lithologies.

**Fig. 14:**
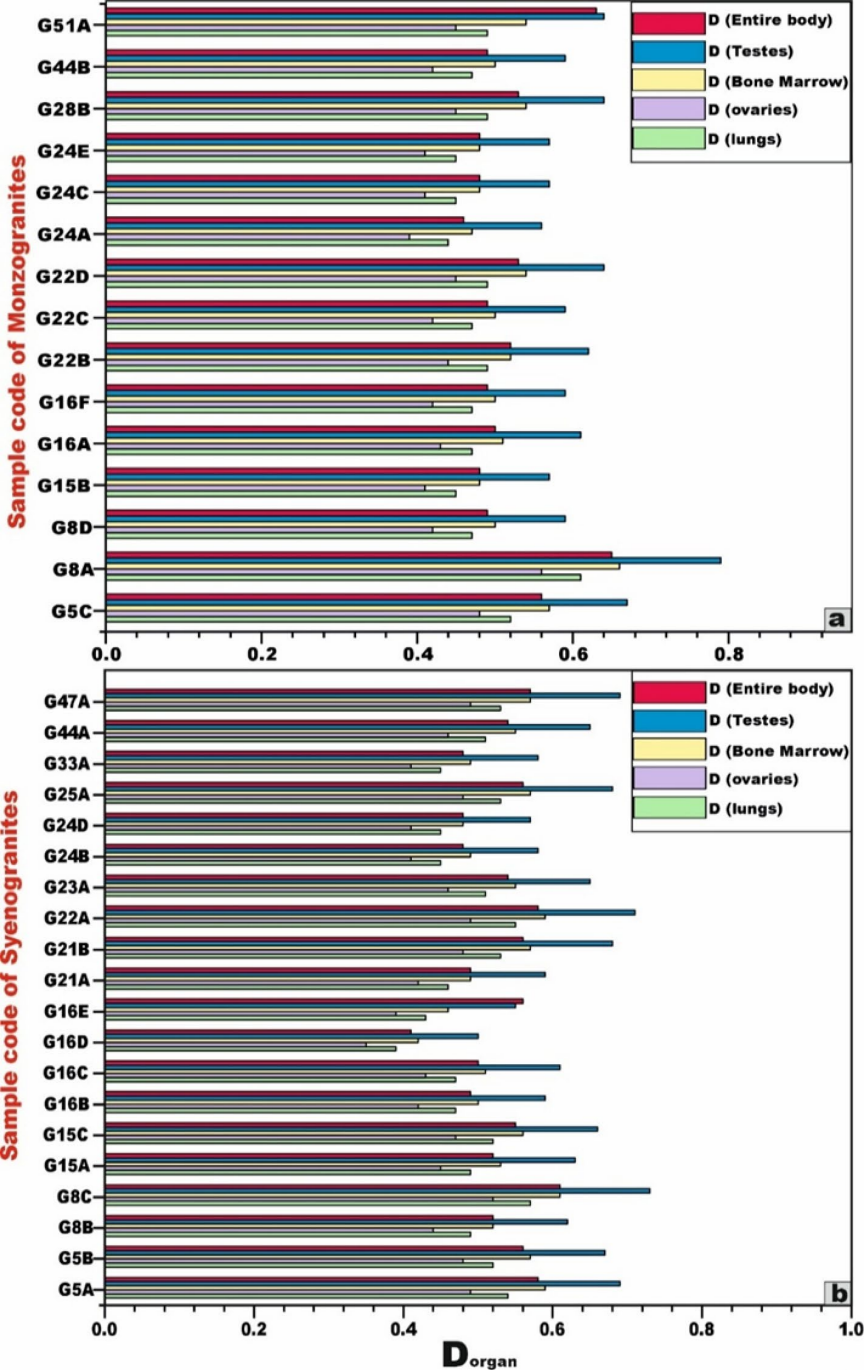
Bar diagram to compare between dose rates (D) of the different organs **(a)** for monzogranites, and **(b)** syenogranites samples from Wadi El-Nabi’ area, Egyptian Nubian Shield.

Concerning the effective dose rates to various organs (D_organ_), the average values for the examined monzogranite and syenogranite samples are as follows: 0.5 mSv for the lungs, 0.4 to 0.5 mSv for the ovaries, 0.5 mSv for the bone marrow, 0.6 mSv for the testes, and 0.5 mSv for the whole body (Table 3). These results demonstrate that the radiation doses received by the evaluated organs from the granitic rocks remain below the internationally accepted annual dose limit of 1.0 mSv^74^ (Fig. 14).

**Fig. 15:**
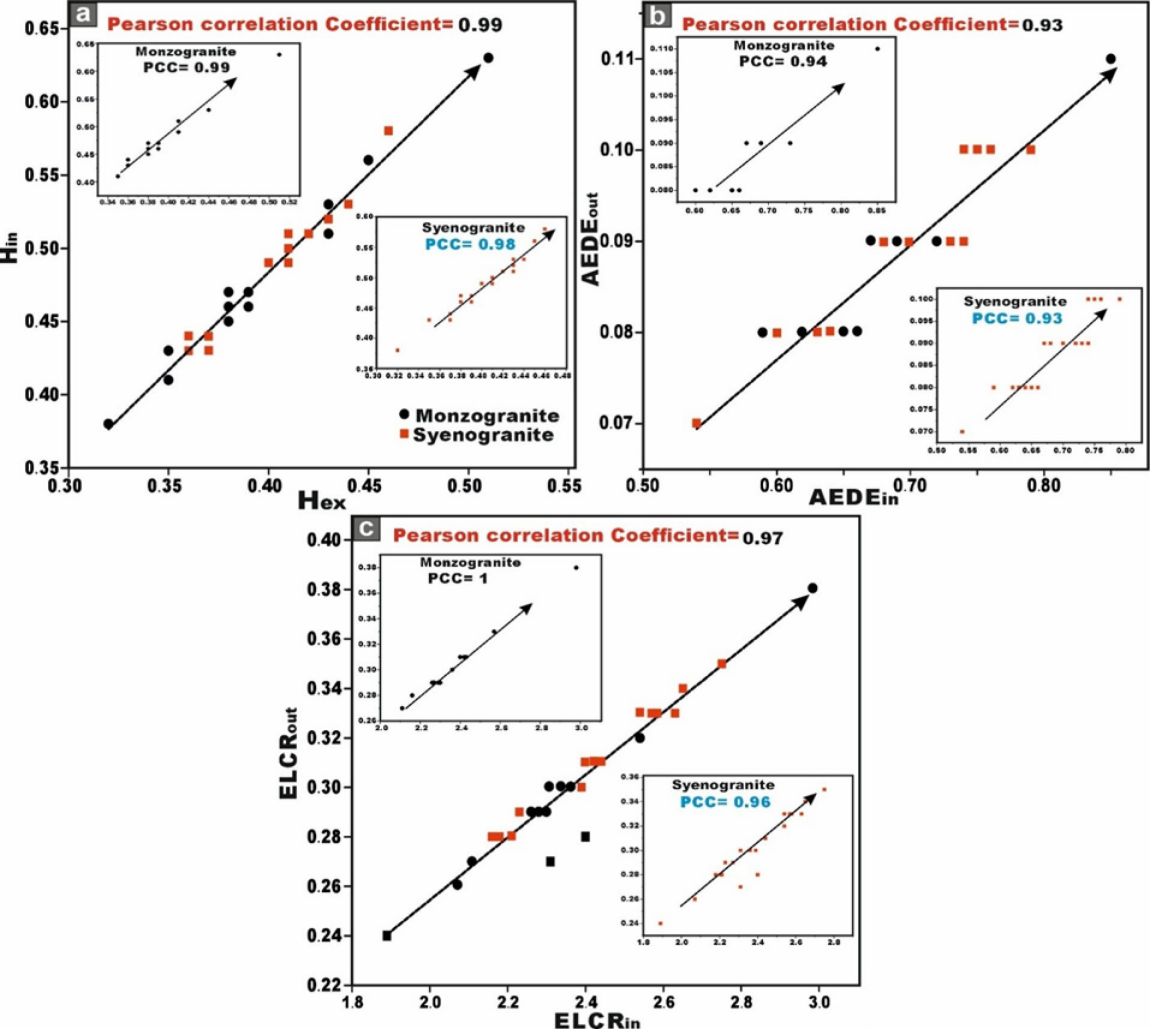
The correlation graphs between **(a)** hazard index indoor (H_in_) and outdoor (H_ex_) (mSv/y), **(b)** Annual effective dose indoor (AEDE_in_) and outdoor (AEDE_out_) (mSv/y), and **(c)** lifetime cancer risk indoor (ELCR_in_) and Outdoor (ELCR_out_) for the studied monzo-syenogranites.

With respect to the relationships among the various radiological hazard indices (Fig. 15), Fig. 15a presents correlation plots illustrating the association between H_in_ and the H_ex_. The analysis indicates an exceptionally strong positive correlation across all granitoids samples (r = 0.99). This robust relationship persists when the lithologies are examined independently, yielding correlation coefficients of r = 0.99 for the monzogranites and r = 0.98 for the syenogranites. Similarly, Fig. 15b depicts the correlation between AEDE_in_ and AEDE_out_, demonstrating a pronounced positive correlation for the entire dataset (r = 0.93), which remains consistently strong for both monzogranites (r = 0.94) and syenogranites (r = 0.93) subsets. Furthermore, Fig. 15c illustrates the relationship between the ELCR_in_ and ELCR_out_, likewise exhibiting a strong positive correlation for all granitoid samples combined (r = 0.97). When assessed separately, the correlation reaches unity for the monzogranites (r = 1.00) and remains markedly strongly positive for the syenogranites (r = 0.96).

**Fig. 16:**
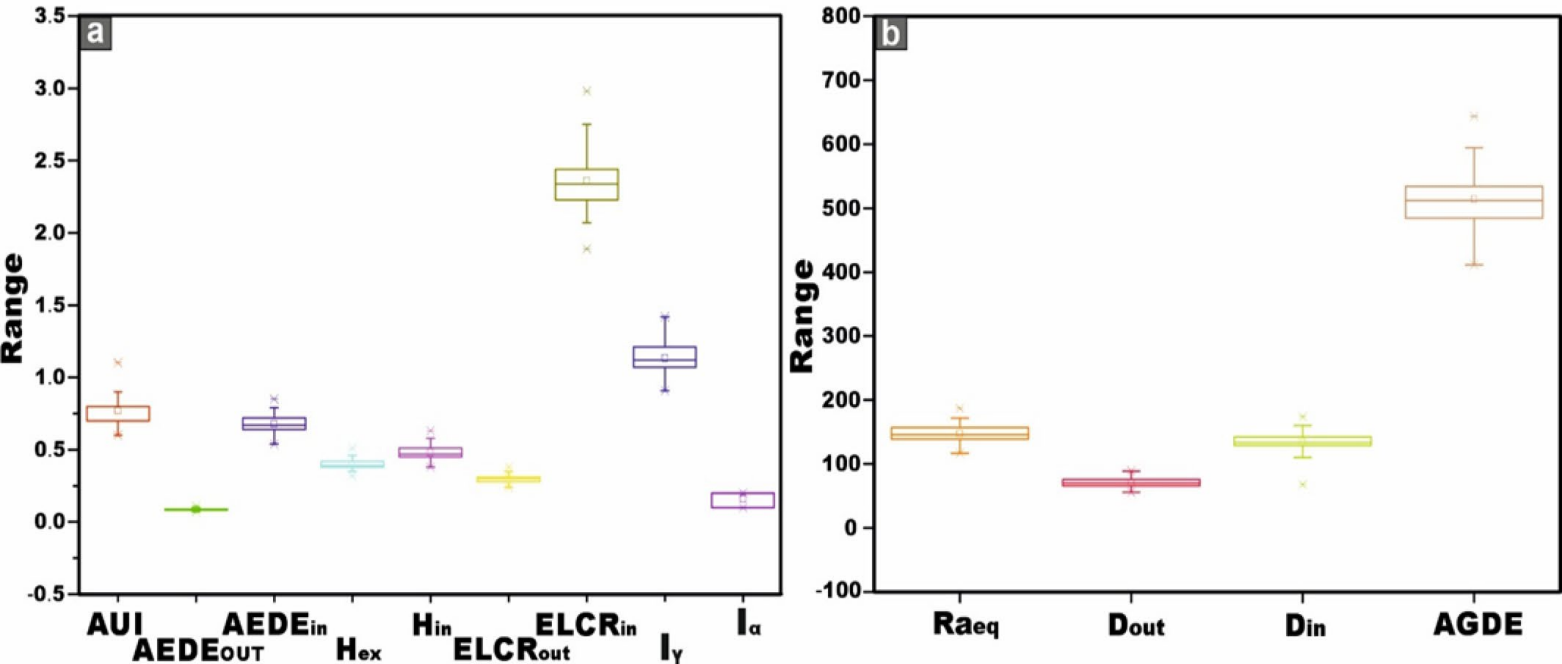
**(a–b)** Box-plot representations illustrating the range, mean, and median values of the radiological hazard indices determined for the analyzed monzo-syenogranite samples.

Table 2 furnishes a comprehensive synthesis of the range, mean, and median values of the radiological hazard indices determined for the analyzed monzogranite and syenogranite samples. These statistical distributions are effectively visualized through an accompanying box-plot representation, providing a clear and integrative depiction of the variability within each lithological group (Fig. 16a, b).

### Radiogenic heat production (RHP) evaluation

The radioactive disintegration of the radionuclides within rocks is a complex process that involves energy release, with a substantial portion of this energy being transformed into heat. The decay chains of ^226^Ra (^238^U), ^232^Th, and ^40^K make significant contributions to this thermal energy compared to other radioisotopes present in the rock samples. The heat generated per second from a rock volume due to the ongoing radioactive disintegration is referred to as radiogenic heat production (RHP). This parameter is directly influenced by the geochemical composition and characteristics of the rock under investigation, and it can be calculated as follows ^75,76^.

## RHP μWm^-^^3^= (9.52C_U_ + 2.56C_Th_ + 3.48C_K_) ƥ/10^5^

Where ρ is the bulk density of the rock, C_U_ and C_Th_ are the U and Th concentrations in weight ppm, and C_K_ is the K concentration in weight %. These concentrations were multiplied by the heat generation constants (the amount of heat released per gram of U, Th, and K per unit time).

Examining radiogenic heat production (RHP) is a crucial aspect of our study, as it links the elemental concentrations of U, Th, and K to their heat-generating potentials. Rocks exhibiting RHP values exceeding 4 μW/m^3^ are classified as having high RHP potential^77^. Conversely, those with RHP values below 2 μW/m^3^ are deemed to possess a low RHP rate. Additionally, rocks with RHP values ranging from 2 to 4 μW/m^3^ are characterized as having a moderate RHP.

Using the data presented in Table 4, we converted the activity concentration from Bq/kg to ppm utilizing specific equations [1 ppm of ^226^Ra (^238^U) = 11.1 Bq/kg, 1 ppm of ^232^Th = 4.06 Bq/kg, and 1 ppm of ^40^K = 313 Bq/kg]^78^. The concentrations of eRa (eU) within the study area ranged from 1.89 to 4.05 ppm in monzogranites and 1.98 to 3.69 ppm in syenogranites, with respective mean values of 2.65 (± 0.5) ppm and 2.78 (± 0.34) ppm. Similarly, eTh concentrations varied between 5.67 and 11.58 ppm in monzogranites and 6.89 to 10.34 ppm in syenogranites, averaging 8.27 (± 1.35) ppm and 8.52 (± 0.98) ppm, respectively. Potassium (K) concentrations were observed to span from 2.59 to 3.16% in monzogranites and from 2.22 to 3.54% in syenogranites, with average values of 2.82% (± 0.15) and 2.84% (± 0.28), respectively (Table 4). From these radioelement concentrations, the Radiogenic Heat Production (RHP) values were calculated, ranging between 1.17 and 2.14 μW/m^3^ for monzogranites and 1.20 and 1.93 μW/m^3^ for syenogranites, with mean values of 1.52 (± 0.22) μW/m^3^ and 1.57 (± 0.17) μW/m^3^, respectively (Table 4). These results indicate that the studied rocks exhibit a low potential for radiogenic heat production and their limited contribution to geothermal energy sources.

**Table 4:** Transformation of ^226^Ra, ^232^Th, and ^40^K from Bq/kg into ppm values for calculation of radiogenic heat production (RHP μW/m^3^).

Sample code	Rock Type	eU ppm	eTh ppm	K%	RHP	RHP μW/m^3^
G5C	Monzogranite	2.97	10.09	2.89	0.0017	1.73
G8A	Monzogranite	4.05	11.57	3.13	0.0021	2.14
G8D	Monzogranite	2.88	6.89	2.79	0.0014	1.48
G15B	Monzogranite	2.43	7.142	2.69	0.0014	1.37
G16A	Monzogranite	2.43	8.374	2.78	0.0015	1.47
G16F	Monzogranite	2.43	7.88	2.79	0.0014	1.43
G22B	Monzogranite	2.70	7.88	2.91	0.0015	1.51
G22C	Monzogranite	2.61	7.63	2.78	0.0015	1.46
G22D	Monzogranite	2.61	9.35	2.82	0.0016	1.58
G24A	Monzogranite	2.07	7.88	2.59	0.0013	1.32
G24C	Monzogranite	2.07	7.63	2.74	0.0013	1.32
G24E	Monzogranite	1.89	5.66	3.15	0.0012	1.17
G28B	Monzogranite	2.79	8.86	2.80	0.0016	1.59
G44B	Monzogranite	2.70	8.37	2.66	0.0015	1.52
G51A	Monzogranite	3.15	8.86	2.72	0.0017	1.68
G5A	Syenogranite	2.97	7.14	3.53	0.0016	1.59
G5B	Syenogranite	2.88	9.85	2.91	0.0017	1.69
G8B	Syenogranite	2.70	7.38	2.99	0.0015	1.48
G8C	Syenogranite	3.69	10.09	3.01	0.0019	1.93
G15A	Syenogranite	3.06	7.63	2.87	0.0016	1.59
G15C	Syenogranite	2.88	8.86	3.06	0.0016	1.64
G16B	Syenogranite	2.70	7.88	2.71	0.0015	1.49
G16C	Syenogranite	2.52	8.37	2.77	0.0015	1.49
G16D	Syenogranite	1.98	6.89	2.30	0.0012	1.20
G16E	Syenogranite	2.43	8.62	2.22	0.0014	1.43
G21A	Syenogranite	2.70	7.38	2.70	0.0015	1.46
G21B	Syenogranite	3.15	9.35	2.95	0.0017	1.74
G22A	Syenogranite	3.60	9.35	2.94	0.0019	1.85
G23A	Syenogranite	2.79	8.86	2.87	0.0016	1.60
G24B	Syenogranite	2.34	8.37	2.62	0.0014	1.43
G24D	Syenogranite	2.43	8.86	2.44	0.0015	1.47
G25A	Syenogranite	2.79	9.35	3.10	0.0017	1.66
G33A	Syenogranite	1.98	7.63	2.89	0.0013	1.31
G44A	Syenogranite	3.06	8.12	2.96	0.0016	1.63
G47A	Syenogranite	2.97	10.34	2.92	0.0016	1.75
Min	Monzogranite	1.89	5.67	2.59	0.0011	1.17
	Syenogranite	1.98	6.89	2.22	0.0012	1.20
Max	Monzogranite	4.05	11.58	3.16	0.0021	2.14
	Syenogranite	3.69	10.34	3.54	0.0019	1.93
Avg	Monzogranite	2.65	8.27	2.82	0.0015	1.52
	Syenogranite	2.78	8.52	2.84	0.0016	1.57
SD	Monzogranite	0.5	1.35	0.15	0.0002	0.22
	Syenogranite	0.34	0.98	0.28	0.0002	0.17

**N.B** 1ppm of ^226^Ra = 11.1 Bq/kg, 1ppm of ^232^Th = 4.06 Bq/kg, and 1ppm of ^40^K = 313 Bq/kg^78^.

**Fig. 17:**
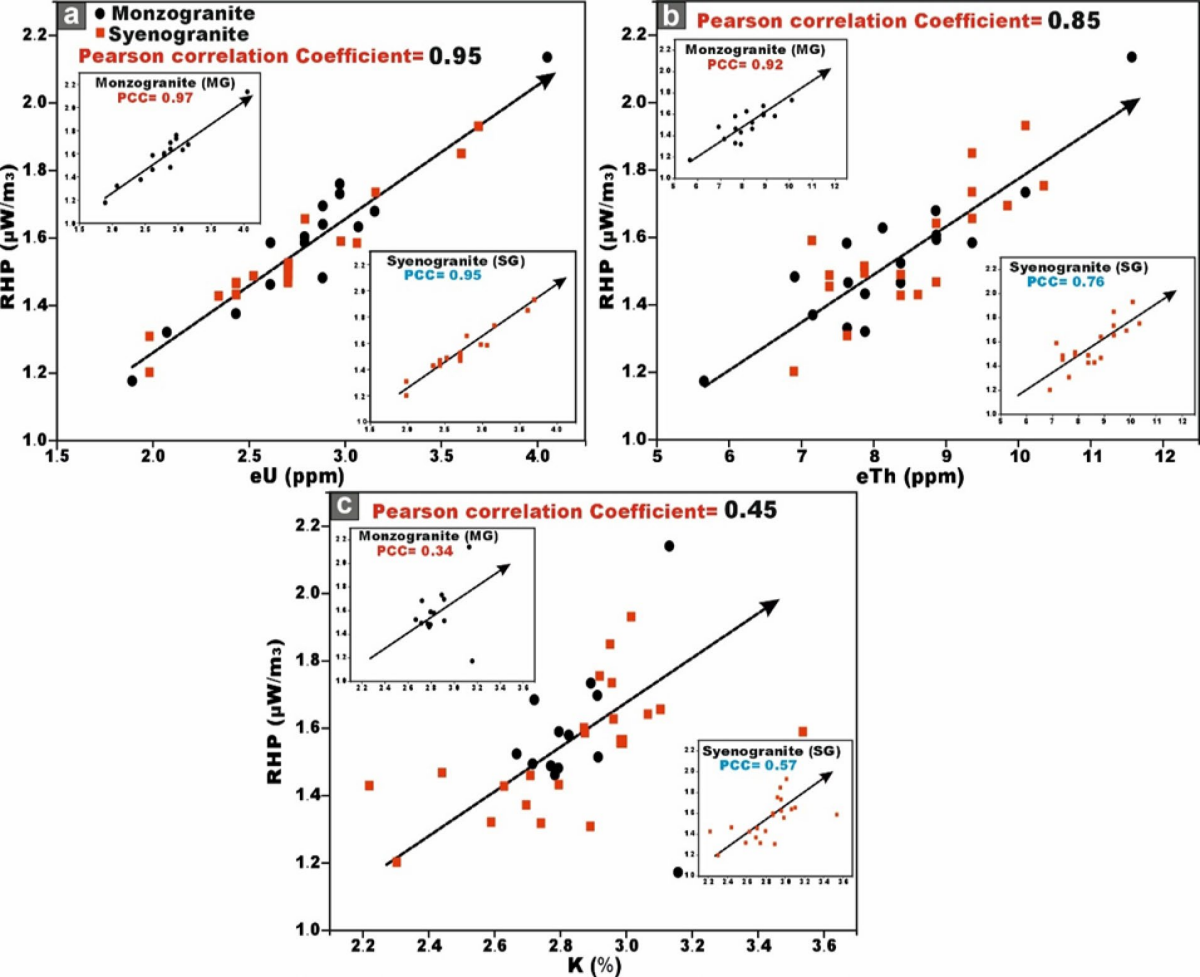
Correlations between the activity concentrations of the principal radioelements (ppm) and the radiogenic heat production (RHP) for the investigated monzo-syenogranite samples from the Wadi El-Nabi’ area. **(a)** eU (ppm) and RHP, **(b)** eTh (ppm) and RHP, and **(c)** K (%) and RHP.

Figure 17a–c illustrates the correlations between the activity concentrations of the principal radioelements (ppm) and the radiogenic heat production (RHP) for all investigated monzogranite and syenogranite samples. In Fig. 17a, a compellingly strong positive linear correlation is observed between eU (ppm) and RHP (r = 0.95) for the entire monzo-syenogranite dataset. When the lithologies are considered separately, this relationship remains exceptionally robust, yielding correlation coefficients of r = 0.97 for the monzogranites and r = 0.95 for the syenogranites. Such consistently high correlations underscore the dominant contribution of uranium to radiogenic heat generation across both rock types. Moreover, Fig. 17b elucidates the association between eTh (ppm) and RHP, which likewise demonstrates a strong positive linear correlation. Upon separation of the samples, the monzogranites exhibit a very strong correlation (r = 0.92), while the syenogranites display a comparably strong relationship (r = 0.76). This further reinforces thorium’s substantial, though secondary, role in heat production. In contrast, Fig. 17c reveals that the relationship between K (%) and RHP is comparatively weak. The correlation coefficient for the combined dataset is r = 0.45, as the monzogranites yield a weak correlation (r = 0.34) and the syenogranites exhibit a moderate correlation (r = 0.57). This attenuated relationship highlights potassium’s relatively minor contribution to radiogenic heat production when juxtaposed with the markedly stronger influence of uranium and thorium.

### Statistical analysis

**Fig. 18:**
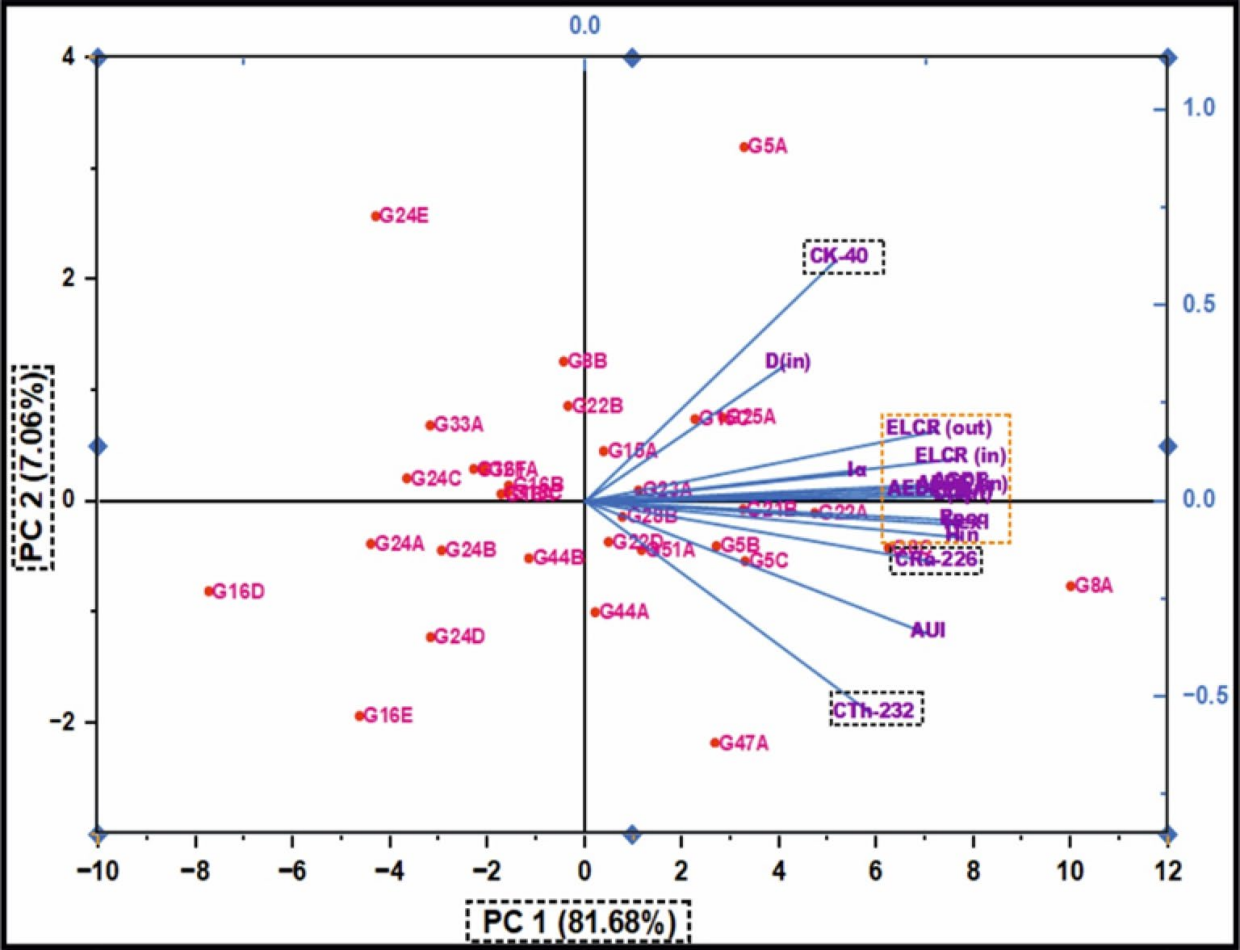
Principal component analysis (PC1 and PC2) for radiological data for the studied monzo-syenogranites from Wadi El-Nabi’ area, Egyptian Nubian Shield.

The levels of natural radioactivity in the study area were systematically evaluated through multivariate statistical analyses employing Origin and STATGRAPHICS Centurion XVI software. The specific activity concentrations of radionuclides were determined for all samples and subsequently compared to global reference values (Fig. 8a–c). In addition, the radiological hazard indices of the investigated granitic rocks were assessed against internationally recommended standards (Fig. 10). Furthermore, the Pearson correlation coefficients (Table 5) and principal component analysis (PCA) (Fig. 18) were computed using Origin software to elucidate the underlying relationships among radionuclides and radiological parameters.

**Table 5:** Pearson correlation between natural radionuclides and the radiological hazards coefficients for the studied monzo-syenogranites from Wadi El-Nabi’, Egyptian Nubian Shield.

Variables	^226^Ra	^232^Th	^40^K	Ra_eq_	D_out_	D_in_	AUI	AEDE_out_	AEDE_in_	H_ex_	H_in_	ELCR_out_	ELCR_in_	I_γ_	I_α_	AGDE
^226^Ra	1															
^232^Th	0.68	1														
^40^K	0.46	0.13	1													
Ra_eq_	0.90	0.79	0.65	1												
D_out_	0.88	0.76	0.71	0.99	1											
D_in_	0.43	0.32	0.36	0.48	0.48	1										
AUI	0.86	0.89	0.37	0.91	0.89	0.39615	1									
AEDE_out_	0.85	0.67	0.68	0.93	0.94	0.38	0.82	1								
AEDE_in_	0.89	0.74	0.72	0.99	0.99	0.499	0.88	0.93	1							
H_ex_	0.90	0.79	0.64	0.99	0.99	0.48	0.91	0.92	0.99	1						
H_in_	0.95	0.78	0.59	0.99	0.98	0.48	0.92262	0.92	0.98	0.99	1					
ELCR_out_	0.81	0.66	0.66	0.91	0.91	0.79	0.79	0.81	0.92	0.91	0.89	1				
ELCR_in_	0.88	0.71	0.71	0.98	0.98	0.64	0.86	0.90	0.98	0.97	0.96	0.97	1			
I_γ_	0.88	0.76	0.71	0.99	0.99	0.47	0.89	0.94	0.99	0.99	0.98	0.91	0.98	1		
I_α_	0.74	0.38	0.49	0.68	0.68	0.26	0.58	0.65	0.67	0.67	0.70	0.58	0.65	0.68	1	
AGDE	0.89	0.71	0.73	0.99	0.99	0.46	0.86	0.94	0.99	0.98	0.97	0.89	0.97	0.99	0.68	1

### Pearson correlation coefficient

This study employs Pearson correlation analysis to identify associations and linear relationships between the activity concentrations of radionuclides and the radiological hazard indices in the examined granite samples, as it serves to unveil the inherent relationships and association strengths between pairs of variables^79^. Table 5 illustrates the Pearson’s correlation coefficient values computed among radioactivity concentrations for radionuclides and diverse radiological health hazard factors encompassing both indoor and outdoor environments.

Using the Pearson correlation coefficient (PC), the linear relationships among the examined variables were categorized into four levels; the first category denotes a weak correlation ranging from 0.00 to 0.39, the second indicates a moderate correlation between 0.40 and 0.69, the third reflects a strong correlation within the range of 0.70 to 0.89, and the fourth corresponds to a very strong correlation from 0.90 up to 1.00 ^79^. Table 5 demonstrates that the interrelationships among the investigated radionuclides, as well as their correlations with the various radiological hazard indices, exhibit consistently positive. These correlations suggest that the radionuclides present in the investigated samples originate from natural sources.

The correlation matrix indicates that ^232^Th exhibits strong positive correlations with Ra_eq_, D_out_, AUI, AEDE_in_, H_ex_, H_in_, ELCR_in_, I_γ_, and AGDE, while showing moderate associations with AEDE_out_ and ELCR_out_, and only weak correlations with D_in_ and I_α_. In contrast, ^4^^0^K displays strong correlations with D_out_, AEDE_in_, ELCR_in_, I_γ_, and AGDE, moderate correlations with Ra_eq_, AEDE_out_, H_ex_, H_in_, ELCR_out_, and I_α_, and weak associations with both D_in_ and AUI. Notably, ^226^Ra maintains exceptionally strong correlations with all radiological hazard indices, reflecting its dominant influence on the overall radiological behavior of the studied granites, while showing only a moderate correlation with D_in_.

### Principal component analysis (PCA)

The principal component analysis (PCA) biplot illustrates the multivariate relationships between radionuclide activity concentrations and radiological hazard parameters in the investigated monzo-syenogranite samples. The first two principal components (PC1 and PC2) together account for 88.74% of the total variance, with PC1 explaining 81.68% and PC2 contributing 7.06% (Fig. 18). PC1 is strongly and positively loaded with ^226^Ra, ^232^Th, Ra_eq_, D_out_, D_in_, AEDE_out_, AEDE_in_, ELCR_out_, ELCR_in_, H_ex_, H_in_, I_γ_, and AGDE. This strong collinearity demonstrates that radiological hazard indices are predominantly controlled by ^226^Ra and ^232^Th activity concentrations, particularly ^226^Ra, which acts as the principal contributor to gamma radiation and dose-related parameters. Conversely, PC2 reveals weak negative loadings for ^40^K, explaining only 7.06% of the variance. The negative loadings indicate that these radionuclides have a limited influence on the overall radiation exposure in the analyzed samples.

The distribution of sample scores reveals that most samples cluster near the origin or along the positive PC1 axis, indicating relatively homogeneous radiological behavior across the study area. However, a limited number of samples deviate toward higher PC1 loadings, possibly associated with hydrothermal alteration corridors, fracture systems, an interpretation consistent with the reduced ^232^Th/^226^Ra ratios.

Overall, the PCA confirms that the radiological risk in the Wadi El-Nabi′ granitoids is dominantly governed by uranium–thorium systematics, with potassium playing a subordinate role, accounting for 88.74% of the total variance in the radiological dataset, underscoring the robustness and reliability of the analysis ^80^.

## Remote sensing analysis

**Fig. 19:**
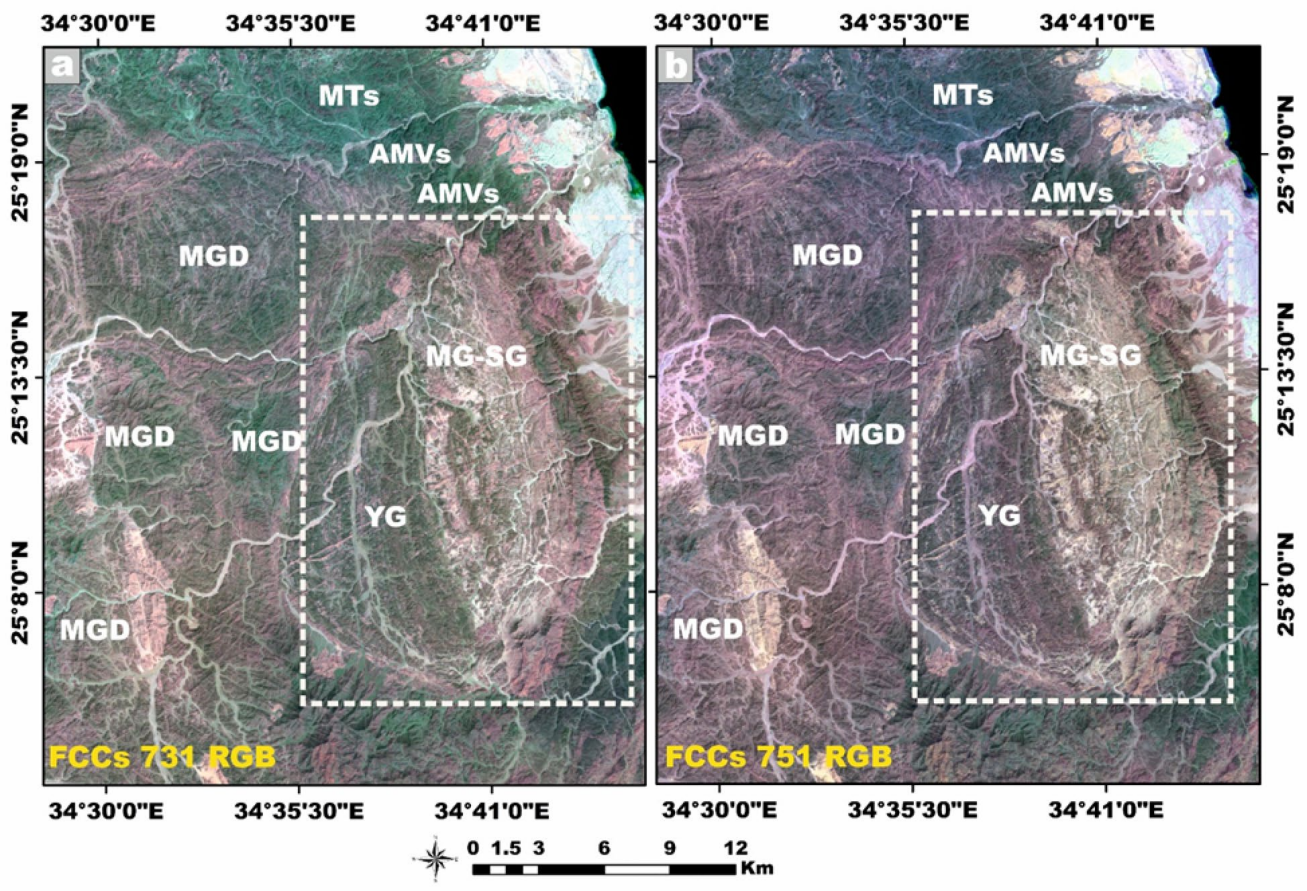
Lithological discrimination using Landsat-9 satellite imagery FCCs in the Wadi El-Nabi’ area. **(a**) FCCs 7, 3, and 1 in RGB and **(b)** FCCs 7,5 and 1 in RGB channels. **Symbols MG-**SG = monzo-syenogranites, YG = younger gabbro, MGD = metagabbro-diorite, AMVs = arc metavolcanics, and MTs = meta tuffs. Created by QGIS v. 3.40.9–Bratislava software; (https://qgis.org/) and ArcGIS Desktop 10.8. https://www.esri.com/en-us/arcgis/products/arcgis-desktop/overview, .

**Fig. 20:**
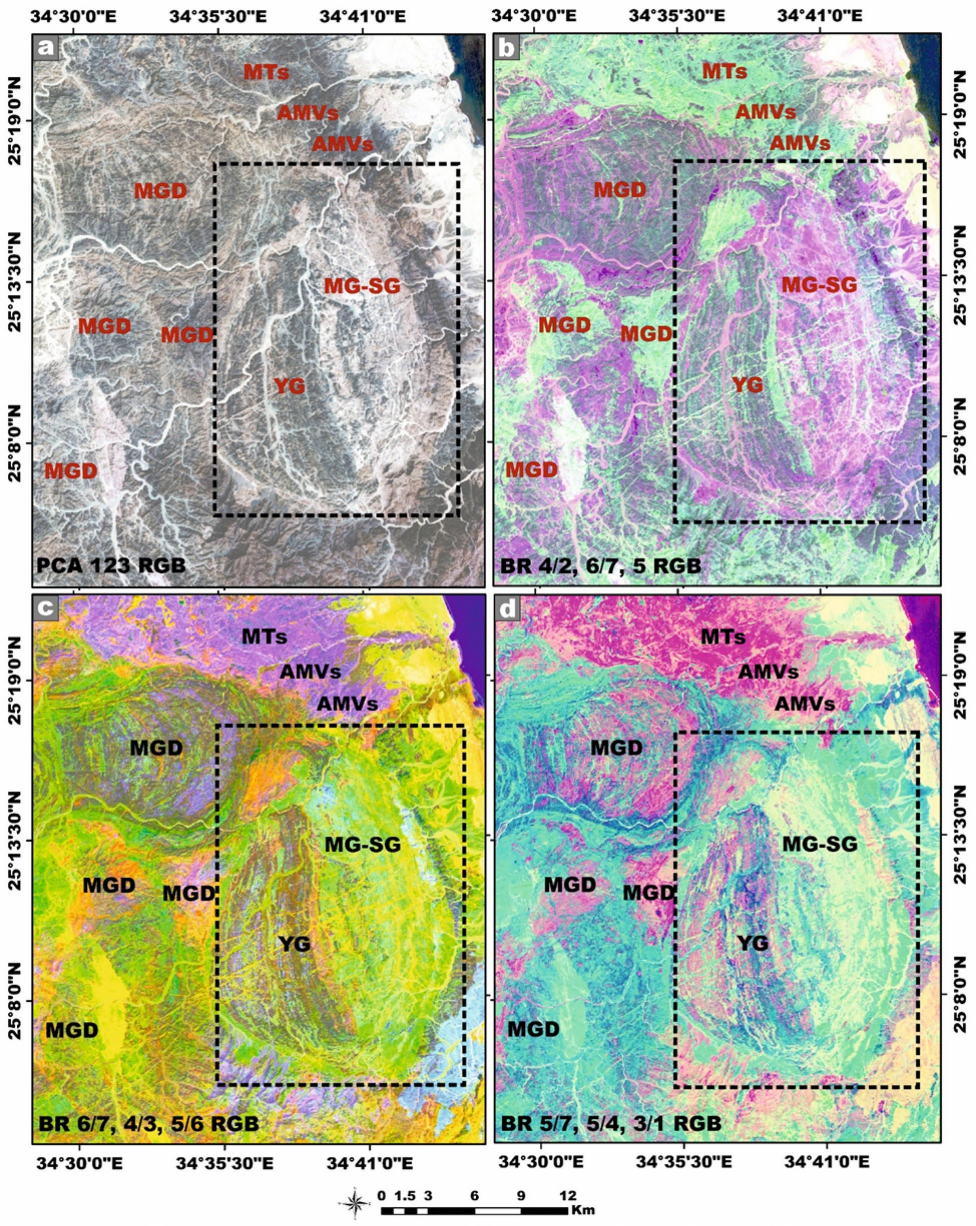
Lithological characterization utilizing Landsat-9 satellite imagery transformation combinations to Wadi El-Nabi’ area **(a)** PCA 1–2-3, **(b)** band ratio (BR) 4/2, 6/7, and 5, **(c)** band ratio (BR) 6/7, 4/3, and 5/6, and **(d)** band ratio (BR) 5/7, 5/4, and 3/1 in RGB channels. Symbols MG-SG = monzo-syenogranites, YG = younger gabbro, MGD = metagabbro-diorite, AMVs = arc metavolcanics and MTs = meta tuffs. Created by QGIS v. 3.40.9–Bratislava software; (https://qgis.org/) and ArcGIS Desktop 10.8. https://www.esri.com/en-us/arcgis/products/arcgis-desktop/overview, .

For effective delineation and differentiation of distinct rock lithologies, Landsat-9 satellite imagery was utilized to enhance the mapping and spectral discrimination of radionuclide-bearing lithologies, with particular emphasis on the El-Igl El-Ahmer monzo-syenogranites. The analysis employed optimized false-color composite band combinations (7, 3, 1 and 7, 5, 1 in RGB) (Fig. 19a, b). These composites significantly improved the segregation of lithological units across the Wadi El-Nabi’ area, enabling clear differentiation of the monzo-syenogranitic assemblage (MG–SG), which is distinctly accentuated within the delineated white square. Additionally, principal component analysis (PCA) was implemented using bands 1, 2, and 3 (Fig. 20a), yielding a markedly improved spectral separation of the various lithological units. This transformation effectively accentuated the monzo-syenogranites, which appear in a distinct pale brownish tonal signature, thereby enhancing their visual detectability and facilitating their discrimination from the surrounding rock assemblages.

Moreover, advanced false-color composite band-ratio techniques, such as (4/2, 6/7, 5), (6/7, 4/3, 5/6), and (5/7, 5/4, 3/1) applied in RGB channels (Fig. 20b–d), exhibited exceptional effectiveness in discriminating the lithological assemblages within the study area. These spectral transformations not only enhanced lithological contrast but also facilitated the precise delineation of granitic bodies. Notably, the monzo-syenogranites were distinctly resolved, manifesting in characteristic fuchsia, pale-green, and turquoise tonal signatures (MG–SG), respectively (Fig. 20b–d).

Indeed, alterations associated with radioactive mineralization often manifest as well-defined hydrothermal alteration zones, characterized by pronounced modifications in mineralogical composition and textural fabric induced by the percolation of thermally elevated, metal-enriched fluids. Among the most diagnostically significant hydrothermal alteration assemblages intimately linked to radioactive deposits are iron oxide, argillic (kaolinization), propylitic (fluoritization), and phyllic (sericitization and silicification) alteration^81,82^.

**Fig. 21:**
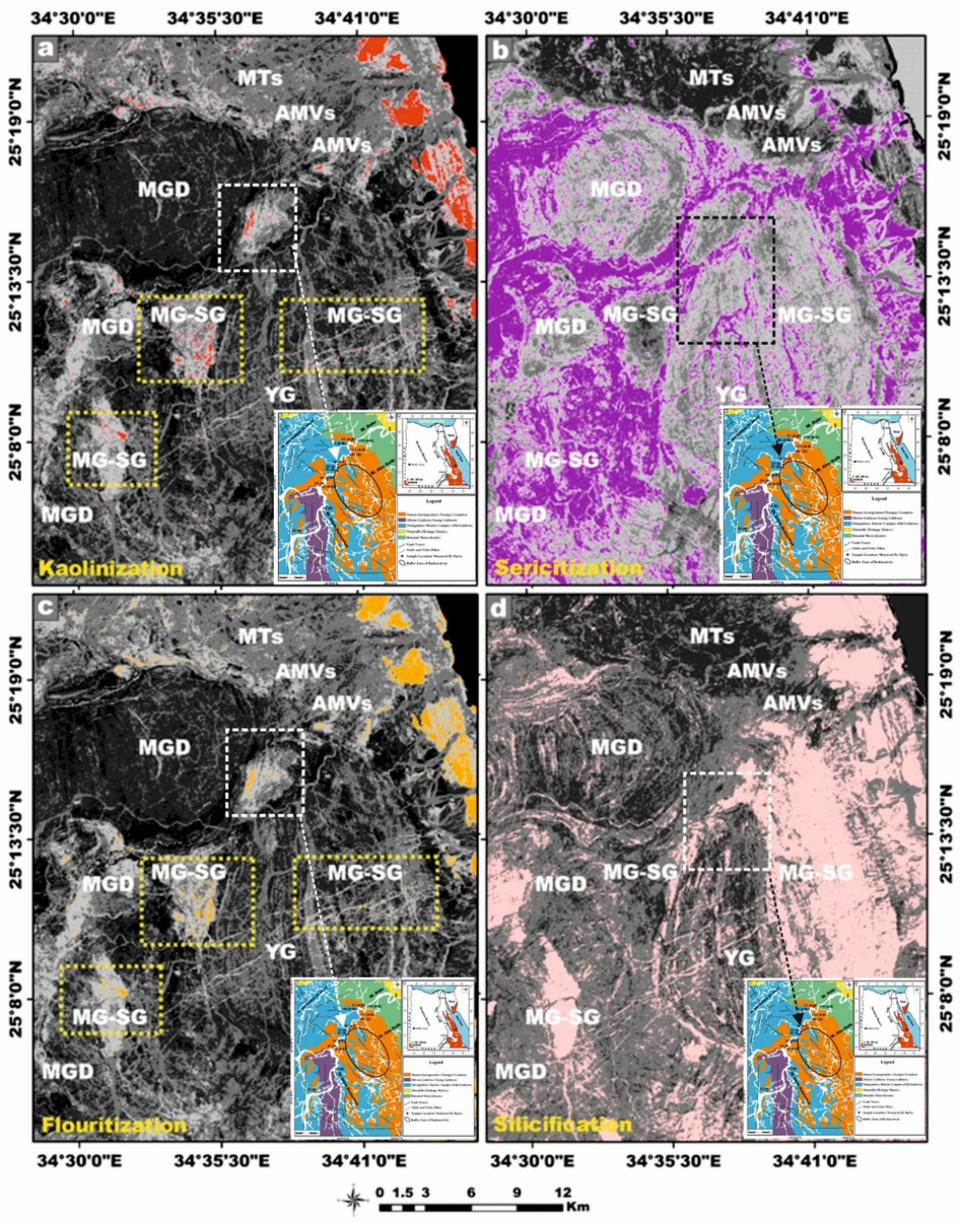
Advanced band ratios (Br) to map alteration zones associated with radioactive mineralization. **(a)** band ratio 6/7 for identifying kaolinization zones **(b)** band ratio (7–5)/(7 + 5) for mapping sericitization alteration zones, **(c)** the ratio (6–7)/(6 + 7) for detection of fluoritization zones, and **(d)** band ratio 5/4 was invaluable for delineating silicification zones. Symbols MG-SG = monzo-syenogranites, YG = younger gabbro, MGD = metagabbro-diorite, AMVs = arc metavolcanics, and MTs = meta tuffs. Created by QGIS v. 3.40.9–Bratislava software; (https://qgis.org/) and ArcGIS Desktop 10.8. https://www.esri.com/en-us/arcgis/products/arcgis-desktop/overview,.

To systematically delineate hydrothermal alteration zones associated with radioactive mineralization, advanced band-ratio image processing techniques were employed with demonstrable efficacy. The 6/7 band ratio proved highly effective in accentuating kaolinization zones (Fig. 21a), whereas the (7–5)/(7 + 5) ratio was instrumental in delineating sericitization alteration assemblages (Fig. 21b). Similarly, the (6–7)/(6 + 7) ratio facilitated the detection of fluoritization zones (Fig. 21c), and the 5/4 band ratio proved invaluable for mapping silicification zones (Fig. 21d). Notably, the majority of these alteration features exhibited a strong spatial correlation with monzo-syenogranitic units, predominantly concentrated within a well-defined buffer zone (Fig. 2).

This interpretation is further corroborated by the observed radioactive correlations, which suggest the pervasive influence of hydrothermal fluids in enhancing radioactive mineralization. The ^232^Th/^226^Ra (^238^U) ratios, markedly lower than the canonical crustal Th/Ra value of ~ 3.5, reflect post-magmatic hydrothermal overprinting and selective uranium enrichment within the host granitoids (Table 1). Similarly, the ^226^Ra (^238^U)/^232^Th ratios, which substantially exceed the average continental-crust ratio (U/Th ≈ 0.25), provide compelling evidence for significant uranium enrichment, likely driven by hydrothermal mobilization and concentration processes within structurally controlled zones of these granitoid units (Table 1).

**Fig. 22:**
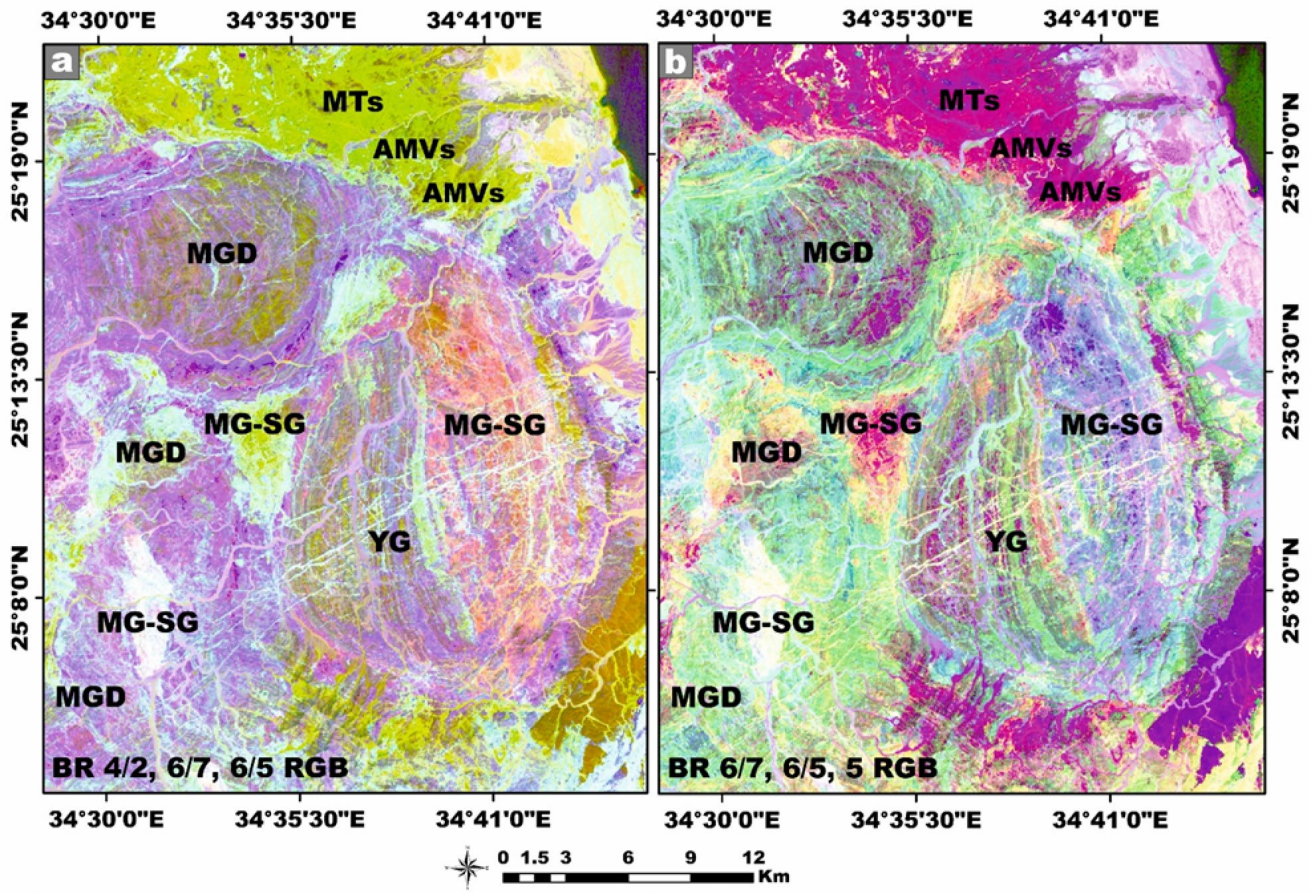
Advanced false-color composites band ratios for detecting radioactive-bearing rocks and Fe-OH-bearing alteration zones in the Wadi El-Nabi’ area. **(a)** FCCs band ratio (BR) 4/2, 6/7, and 6/5, and **(b)** FCCs band ratio (BR) 6/7, 6/5, and 5 in RGB channels. Symbols MG-SG = monzo-syenogranites, YG = younger gabbro, MGD = metagabbro-diorite, AMVs = arc metavolcanics, and MTs = meta tuffs. Created by QGIS v. 3.40.9–Bratislava software; (https://qgis.org/) and ArcGIS Desktop 10.8. https://www.esri.com/en-us/arcgis/products/arcgis-desktop/overview,.

Furthermore, in the present study, advanced false-color composite band-ratio combinations, specifically (4/2, 6/7, 6/5) and (6/7, 6/5, 5) applied across RGB channels, were employed to delineate radioactive-bearing lithologies and Fe-OH-bearing hydrothermal alteration zones (Fig. 22). The results demonstrate a strong spatial correlation between these alteration features and the monzo-syenogranitic units, corroborating previous observations that such iron-rich and hydrothermal alteration assemblages are intimately associated with the host granitic rocks (Fig. 22).

## Comparative assessment of natural radionuclide levels in granitic rocks

**Fig. 23:**
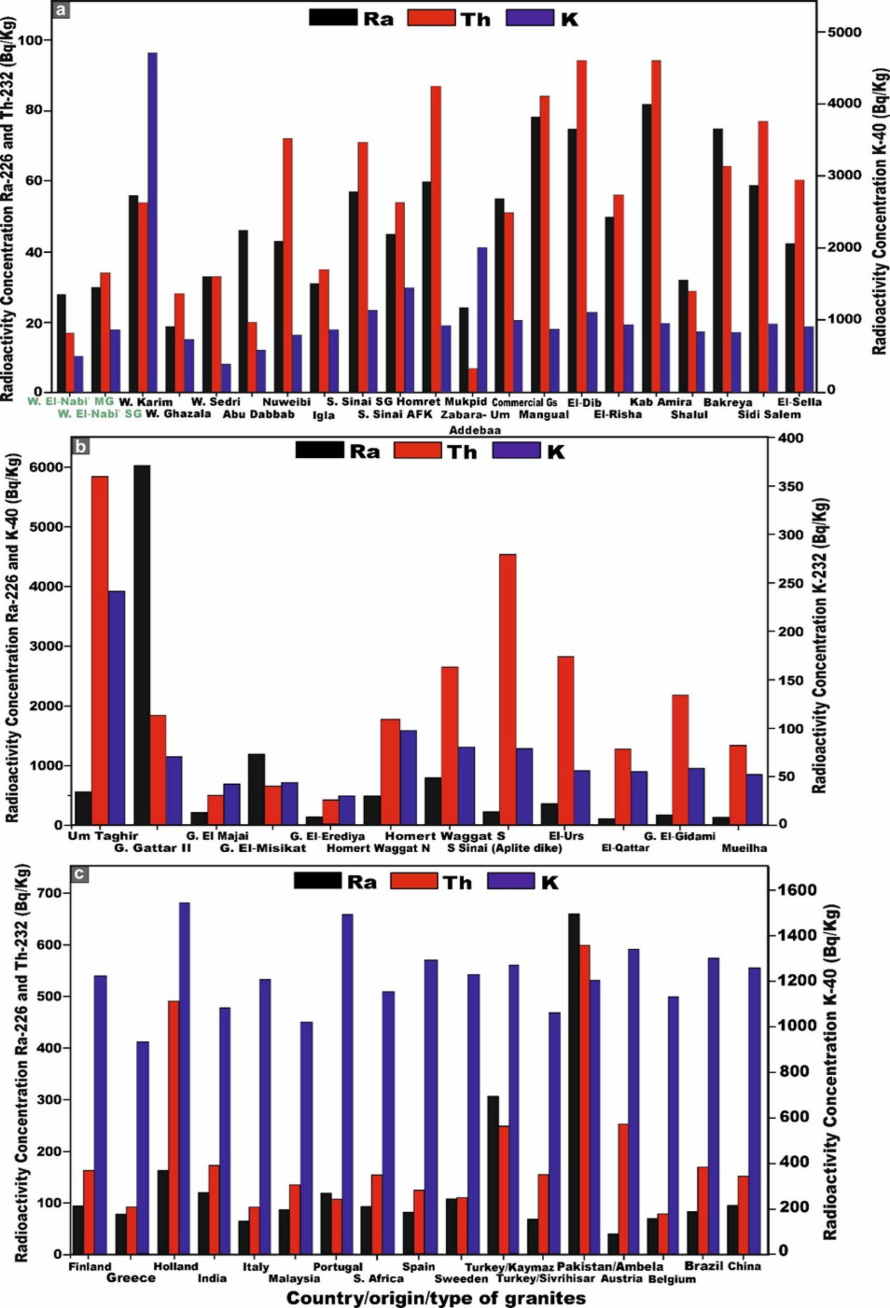
Comparative assessment of the specific activity concentrations of radionuclides (^226^Ra (^238^U), ^232^Th, and ^4^^0^K; Bq/kg) measured in the investigated Wadi El-Nabi’ area relative to those reported for granitic rocks (for building materials). **(a, b)** within the Egyptian Nubian Shield, and **(c)** worldwide. Abbreviations SG = syenogranites; MG = monzogranites.

**Fig. 24:**
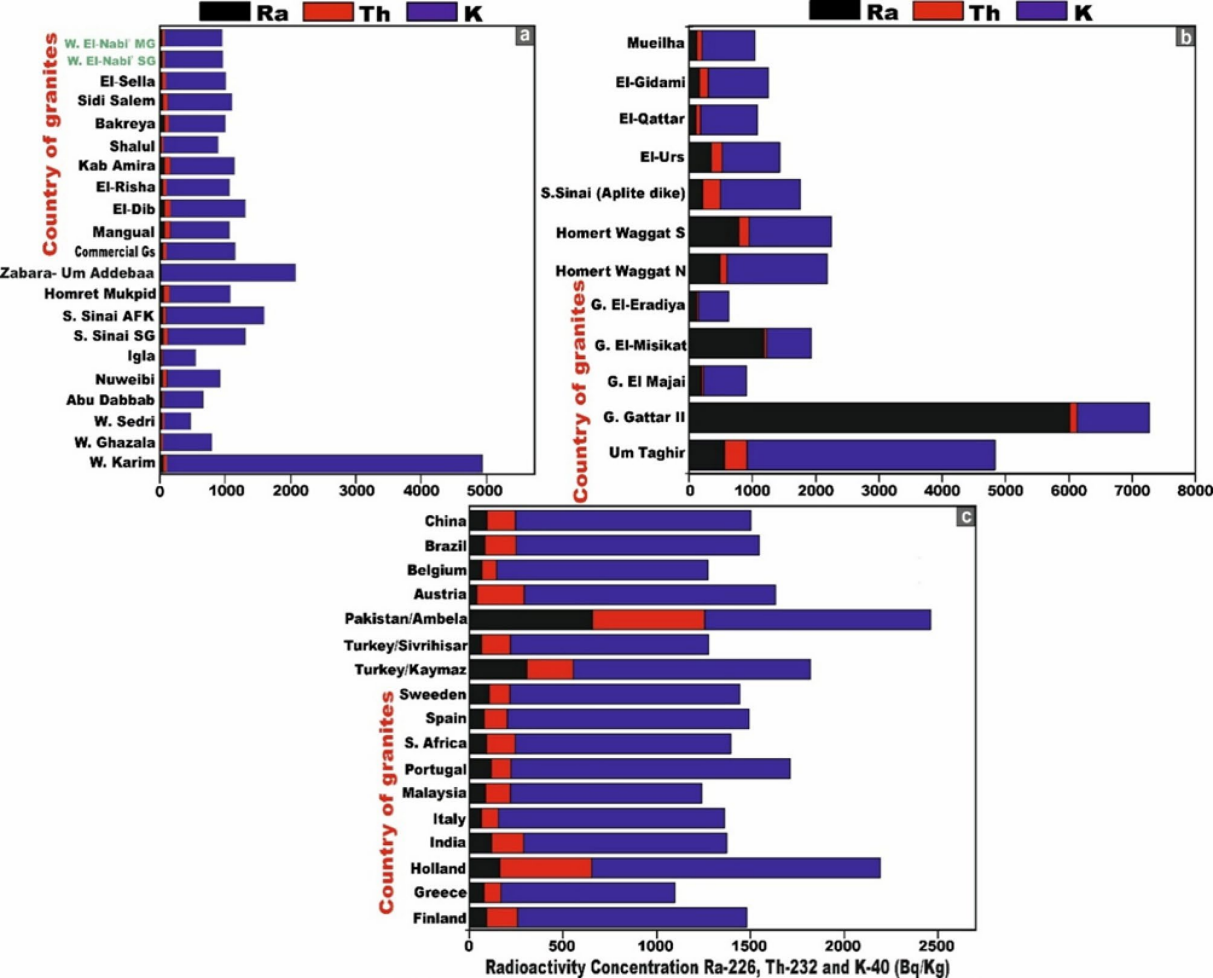
Comparison between the specific activity concentrations of radionuclides (^226^Ra (^238^U), ^232^Th, and ^4^^0^K; Bq/kg) measured in the investigated Wadi El-Nabi’ area relative to those reported for granitic rocks (for building materials) using a stacked bar diagram. (**a**, **b**) within the Egyptian Nubian Shield, and **(c)** worldwide. Abbreviations SG = syenogranites; MG = monzogranites.

Table 6 presents a comprehensive comparative evaluation of the specific activity concentrations of radionuclides (^226^Ra, ^232^Th, and ^4^^0^K), along with their corresponding activity ratios, as determined in the present investigation relative to recently published data for granitic rocks from Egypt and various countries worldwide (Figs. 23 and 24). This comparative framework underscores pronounced regional disparities in radionuclide abundances and facilitates a broader understanding of the radiological characteristics of granitic formations at both local and global scales. Within Egypt, notably elevated ^22^^6^Ra (^238^U) activity concentrations are reported from the Gattar II granite (6018 Bq/kg), whereas exceptionally high ^232^Th contents characterize the Um Taghir area (359 Bq/kg). The highest ^40^K activity within the Egyptian granites is observed at Wadi Karim (4819 Bq/kg). Conversely, comparatively low radionuclide levels are documented for ^226^Ra (^238^U) at Wadi Ghazala (19 Bq/kg), for ^232^Th within the Zabara–Um Addebaa belt (7 Bq/kg), and for ^40^K at Wadi Sedri (403 Bq/kg) (Table 6). On a global scale, granitic rocks from Ambela, Pakistan, exhibit markedly high activity concentrations of both ^22^^6^Ra (^238^U) (659 Bq/kg) and ^232^Th (598 Bq/kg), while the highest reported ^4^^0^K levels are recorded in granites from Holland. In contrast, lower activity concentrations are observed for ^226^Ra (^238^U) in Austria (40 Bq/kg), for ^232^Th in Belgium (77 Bq/kg), and for ^40^K in granites from Greece (929 Bq/kg) (Table 6) ( Figs. 23 and 24).

**Table 6:** Comparison of activity concentration (^226^Ra (^238^U), ^232^Th, and ^40^K) and their ratios of the present study with other data from different granites from Egypt and other countries.

Country/origin/type of granites	No. of samples	Ra-226 (Bq/kg)	Th-232 (Bq/kg)	K-40 (Bq/kg)	Ra-226 /K-40	Th-232/K-40	Ra-226/Th-232	References
Egypt/Wadi Karim	10	56	54	4819	0.01	0.01	1.03	^13^
Egypt/Um Taghir	39	558	359	3918	0.14	0.09	1.55	^13^
Egypt/Gable Gattar II	10	6018	113	1140	5.27	0.09	53.25	^84^
Egypt/Gable El Majai	10	198	30	681	0.29	0.04	6.6	^83^
Egypt/Gable El-Misikat	9	1184	40	705	1.67	0.05	29.6	^83^
Egypt/Gable El-Eradiya	10	126	25	480	0.26	0.05	5.04	^83^
Egypt/Homert Waggat North	10	489	109	1590	0.30	0.06	4.48	^83^
Egypt/Homert Waggat South	10	787	163	1302	0.60	0.12	4.82	^84^
Egypt/Nubian Shield /Wadi El-Nabi’ Area (Syenogranites)	20	31	35	890	0.03	0.03	0.89	Present study
Egypt/Nubian Shield /Wadi El-Nabi’ Area (Monzogranites)	15	30	34	883	0.03	0.03	0.88	Present study
Egypt/Igla	6	28	17	508	0.05	0.03	1.7	^86^
Egypt/South Sinai (Syenogranites)	10	57	71	1173	0.04	0.06	0.80	^21^
Egypt/South Sinai (Alkali feldspar granite)	10	45	54	1500	0.03	0.03	0.83	^21^
Egypt/South Sinai (Aplite dike)	5	213	279	1268	0.16	0.22	0.76	^21^
Egypt/Wadi Ghazala	7	19	28.2	754	0.03	0.04	0.7	^86^
Egypt/Wadi Sedri	7	33	33	403	0.08	0.08	1	^86^
Egypt/Abu Dabbab (Albite granite)	10	46	20	602	0.07	0.03	2.3	^28^
Egypt/Nuweibi area (Albite granite)	22	43	72	811	0.05	0.09	0.6	^87^
Egypt/Homret Mukpid	7	60	87	934	0.06	0.09	0.7	^86^
Egypt/Zabara‑Um Addebaa belt	10	24	7	2049	0.01	0.003	3.6	^86^
Egypt/Commercial granitic ornamental stones (7 types)	7 types	55	51	1039	0.05	0.05	1.08	^88^
Egypt/Mangual	3	78.4	84.4	903	0.09	0.09	0.9	^85^
Egypt/El-Dib	17	74.9	94.3	1144.1	0.07	0.08	0.8	^85^
Egypt/El-Urs	4	352	173.2	908.4	0.4	0.19	2.03	^85^
Egypt/El-Risha	4	49.8	56.1	958.8	0.05	0.06	0.88	^85^
Egypt/El-Qattar	13	104.4	78.8	892.9	0.11	0.09	1.32	^85^
Egypt/Kab Amira	10	82	94.2	974.7	0.08	0.09	0.87	^85^
Egypt/El-Gidami	7	169	134	951.5	0.18	0.14	1.26	^85^
Egypt/Shalul	4	31.9	29	842.3	0.04	0.03	1.1	^85^
Egypt/Bakreya	4	75	64.4	860.8	0.09	0.07	1.16	^85^
Egypt/Sidi Salem	4	58.6	76.8	982.5	0.06	0.08	0.76	^85^
Egypt/Mueilha	13	121.3	82.2	840	0.14	0.09	1.47	^85^
Egypt/El-Sella	4	42.5	60.1	918.2	0.05	0.07	0.7	^85^
Avg	–	343.6	82.08	1170	0.32	0.07	4.1	
Min	–	19	7	403	0.1	0.003	0.6	–
Max	–	6018	359	4819	5.27	0.22	53.2	–
SD	–	1033	72.49	879.5	0.9	0.04	10	–
Finland	3	94	163	1223	0.07	0.13	0.57	^89^
Greece	49	77	91	929	0.08	0.09	0.84	^94,95^
Holland	1	162	490	1540	0.10	0.31	0.33	^90^
India	4	119	172	1082	0.10	0.15	0.69	^89^
Italy	4	64	91	1206	0.05	0.07	0.70	^91^
Malaysia	1	86	134	1019	0.08	0.13	0.64	^89^
Portugal	1	117	105	1490	0.07	0.07	1.11	^89^
S. Africa	1	92	153	1151	0.07	0.13	0.60	^89^
Spain	1	80	123	1289	0.06	0.09	0.65	^89^
Sweeden	2	107	110	1226	0.08	0.08	0.97	^89^
Turkey/Kaymaz	7	306	248	1266	0.24	0.19	1.23	^92^
Turkey/Sivrihisar	7	67	153	1058	0.06	0.14	0.43	^92^
Pakistan/Ambela	20	659	598	1203	0.54	0.49	1.10	^93^
Austria	1	40	253	1340	0.02	0.10	0.15	^89^
Belgium	1	68	77	1129	0.06	0.06	0.88	^90^
Brazil	14	82	168	1297	0.02	0.12	0.48	^90^
China	8	95	152	1256	0.03	0.12	0.62	^89^
Avg	–	136.17	193	1217.88	0.10	0.14	0.71	–
Min	–	40	77	929	0.02	0.06	0.15	–
Max	–	659	598	1540	0.54	0.49	1.23	–
SD	–	142.6	137.8	151	0.12	0.10	0.28	–

Wadi El-Nabi’ granitoids are interpreted as I-type, island-arc granitoids, which contrasts with several highly radioactive Nubian Shield localities (e.g., El-Missikat, Gattar, Um Taghir, and Wadi Karim) that are commonly associated with evolved A-type granites or strongly hydrothermally altered plutons. These genetic differences directly influence radionuclide enrichment patterns. As well, unlike some Nubian Shield granites that host abundant U-Th-bearing accessory phases (e.g., uraninite, monazite, allanite, and zircon), the investigated rocks exhibit relatively limited enrichment of radioactive accessory minerals, consistent with petrographic observations and their moderate radionuclide contents (except ^4^^0^K shows elevated activities) (Table 6) (Figs. 23 and 24).

**Fig. 25:**
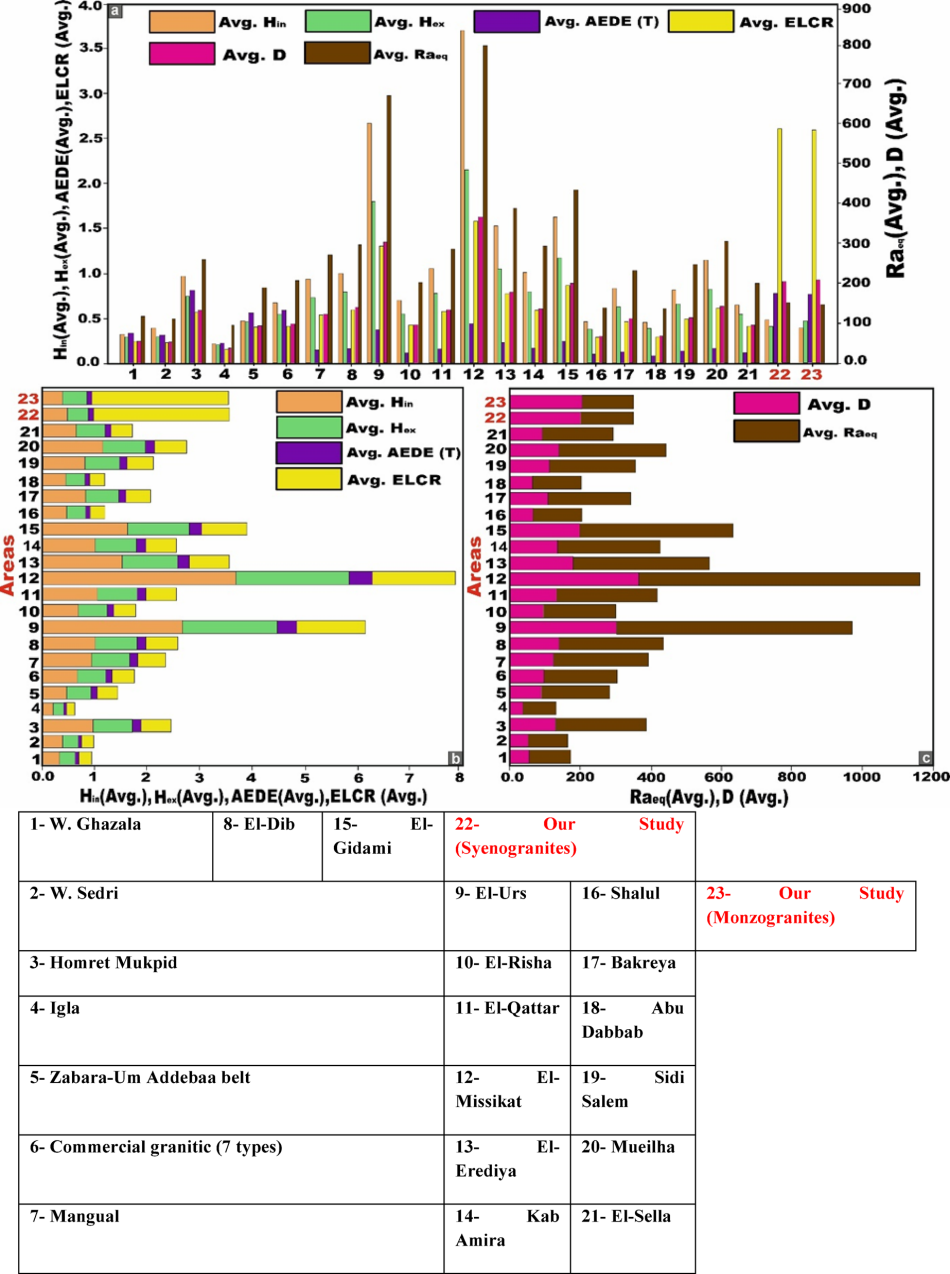
Comparative evaluation of radiation hazard indices for granitic rocks from various localities in Egypt relative to those obtained in the present study from the Wadi El-Nabi’ area. **(a)** Comparison illustrated using column charts, and **(b–c)** representation using stacked bar diagrams.

Figure 25 and Table 7 provide a comprehensive comparative evaluation of the radiation risk parameters associated with granitic rocks from various Egyptian localities, including those examined in the present study from the Wadi El-Nabi’ area. The analysis indicates pronounced spatial variability in radiological hazard indices among the investigated granitic bodies. Notably, the El-Missikat area exhibits the highest recorded values of absorbed dose rate (D), internal hazard index (H_in_), external hazard index (H_ex_), and radium equivalent activity (Ra_eq_), reaching 365.7 nGy/h, 3.69, 2.15, and 764.9 Bq/kg, respectively. In contrast, the lowest values of these parameters are observed in the Igla area, with corresponding values of 36.99 nGy/h, 0.21, 0.20, and 94.76 Bq/kg, respectively (Fig. 25).

**Table 7:** Comparison between radiation hazard indices for some granitic rocks in Egypt and our study (Wadi El-Nabi’ area).

Area	Avg. D (nGy/h)	H_in_	H_ex_	AEDE_out_ [ m Sv y^-1^]	AEDE_in_ [m Sv y^-1^]	Ra_eq_ (Bq/kg)	ELCR × 10^−3^	References
Wadi Ghazala	55.87	0.33	0.3	0.07	0.27	117.59	0.24	^86^
Wadi Sedri	52.93	0.39	0.3	0.06	0.26	112.19	0.23	^86^
Homret Mukpid	132.34	0.97	0.75	0.16	0.65	258.49	0.57	^86^
Igla	36.99	0.21	0.2	0.05	0.18	94.76	0.16	^86^
Zabara-Um Addebaa belt	92.3	0.47	0.46	0.11	0.45	191.42	0.4	^86^
Commercial granitic (7 types)	98	0.67	0.54	0.12	0.48	208	0.42	^88^
Mangual	125.11	0.94	0.73	0.15		268.59	0.54	^85^
El-Dib	139.64	1.01	0.8	0.17		297.9	0.6	^85^
El-Urs	305.44	2.67	1.81	0.37		669.73	1.31	^85^
El-Risha	97.17	0.69	0.55	0.12		203.87	0.42	^85^
El-Qattar	133.34	1.05	0.77	0.16		285.85	0.57	^85^
El-Missikat	365.68	3.69	2.15	0.45		794.93	1.57	^85^
El-Erediya	180.29	1.53	1.05	0.22		387.9	0.77	^85^
Kab Amira	135.7	1.01	0.79	0.17		291.7	0.58	^85^
El-Gidami	199.9	1.63	1.17	0.24		434.36	0.86	^85^
Shalul	67.61	0.46	0.37	0.08		138.18	0.29	^85^
Bakreya	109.69	0.83	0.63	0.13		233.35	0.47	^85^
Abu Dabbab	66.86	0.45	0.37	0.08		138.1	0.29	^85^
Sidi Salem	114.74	0.82	0.66	0.14		244.11	0.49	^85^
Mueilha	140.96	1.15	0.82	0.17		303.49	0.61	^85^
El-Sella	94.52	0.65	0.54	0.12		199.19	0.41	^85^
Our Study (Syenogranites)	203	0.48	0.4	0.09	0.68	149	2.6	–
Our Study (Monzogranites)	207	0.39	0.47	0.09	0.67	146	2.6	–

## Conclusions

It is crucial to provide comprehensive data on the concentrations and distributions of natural radioisotopes in any given area, particularly in regions containing granitic rocks. This information is vital for monitoring environmental contamination resulting from radioactivity, as exposure to radiation poses significant risks to human health. Establishing such baseline data not only aids in assessing current radiological conditions but also serves as a critical reference for tracking potential changes over time. Moreover, this data provides valuable insights for policymakers, enabling informed decisions to mitigate radiation-related hazards and protect public health.

The Wadi El-Nabi’ area has long been recognized as a promising site for mineral exploration, hosting numerous excavation sites for both historical and modern mining and quarrying activities. Additionally, the region serves as a popular open safari destination, attracting visitors and tourists year-round. Given its dual significance as a hub for resource extraction and tourism, it was imperative to focus on evaluating the radioactivity levels and associated risk parameters of the granitic rocks within the study area, which spans approximately 55 km^2^ and forms the core of this region.

**Fig. 26:**
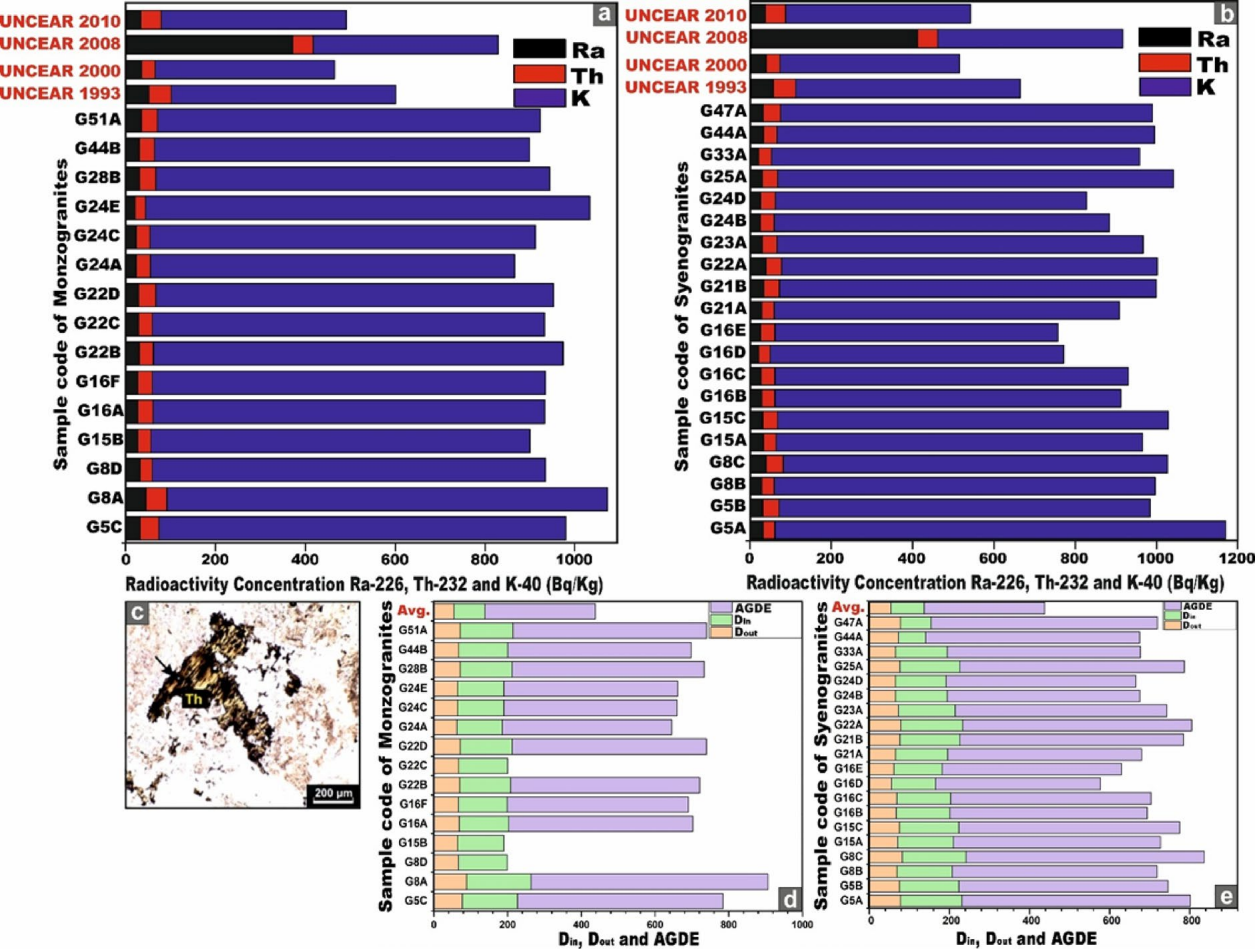
**(a, b)** Plots of the specific activity concentrations ^226^Ra, ^232^Th, and ^40^K (Bq/kg) using stacked bar diagrams for monzogranites and syenogranites samples, **(c)** occurrence of thorite mineral (Th) within syenogranite**, (d and e)** comparative evaluation of some radiation hazard indices for monzogranites and syenogranites, from the Wadi El-Nabi’ area, Egyptian Nubian Shield using stacked bar diagrams.

The activity concentrations of the naturally occurring radionuclides ^22^^6^Ra, ^232^Th, and ^4^^0^K were quantified through detailed radiometric analyses of thirty-five monzo-syenogranite samples collected from surface outcrops in the Wadi El-Nabi′ area (Fig. 26a, b). The results indicate that the mean activity concentrations of ^226^Ra, ^232^Th, and ^40^K in the syenogranite samples are marginally higher than those recorded in the monzogranite counterparts (Fig. 26a, b). This discrepancy is primarily attributed to the comparatively higher abundance of thorium- and potassium-bearing minerals (e.g., thorite, monazite, K-feldspar, and biotite) within the syenogranitic facies, reflecting its more evolved mineralogical composition relative to the monzogranitoids (Fig. 26c).

The activity levels of ^232^Th and ^40^K exceed the internationally recommended reference values. In contrast, the measured activity concentrations of ^226^Ra generally remain below the accepted global reference level, although localized anomalies with relatively elevated values were detected in a few sampling sites. These local enrichments are interpreted to reflect structural controls and late- to post-magmatic processes rather than a regional-scale uranium enrichment. The latter are preferentially concentrated in localized zones, commonly along fractures (NW-SE and N-S structural trends), alteration halos, and highly jointed domains, reflecting the role of late-magmatic and post-magmatic hydrothermal processes in redistributing and concentrating radioactive elements. This interpretation is further supported by the low ^232^Th/^226^Ra (^238^U) ratios and by remote sensing results that delineate hydrothermal alteration zones (e.g., kaolinization, sericitization, and silicification), spatially associated with the granitic bodies.

In addition to quantifying the activity concentrations of the examined radioisotopes (^226^Ra, ^232^Th, and ^40^K), a comprehensive assessment of radiological hazard indices was undertaken. The calculated values for various parameters, including D_out_, D_in_, AEDE_out_, AEDE_in_, ELCR_out_, ELCR_in_, I_γ_, and AGDE (Fig. 26d and e), were found to surpass the recommended reference levels for both monzogranites and syenogranite, especially in the buffer zones. Conversely, the values for Ra_eq_, H_ex_, and H_in_ fell below the established standard levels.

Consequently, granitic rocks within the Wadi El-Nabi’ area exhibit elevated radiological levels that, in certain localized zones – particularly within the identified buffer areas – exceed recommended safety limits for workers engaged in mining and quarrying operations. Moreover, granites from these high-activity zones are considered unsuitable for use as construction and ornamental building materials, as their utilization may pose potential long-term radiological risks to human health and public safety.

This study establishes a foundational baseline for future research, offering critical data to support informed decision-making. We strongly recommend integrating the present map (Fig. 2) into broader efforts to map natural radioactivity across Egypt, enhancing the understanding of radiological profiles in the region.

## Data Availability

All data generated and analysed during this study are included in this published article and its supplementary information files.
